# Elevated [CO_2_ ] Affected Fluctuating Light Acclimation in Cucumber Plants by Changes in Specific Leaf Area and Photosynthetic Efficiency

**DOI:** 10.1111/ppl.70436

**Published:** 2025-08-05

**Authors:** Samikshya Shrestha, Sarah R. Berman, Joke Oosterkamp, Leo F. M. Marcelis, Elias Kaiser, Silvere Vialet‐Chabrand

**Affiliations:** ^1^ Horticulture and Product Physiology, Plant Science Group Wageningen University and Research Wageningen the Netherlands; ^2^ Research Institute of Agriculture and Life Sciences Seoul National University Seoul Republic of Korea

**Keywords:** acclimation, CO_2_, cucumber, fluctuating light, photosynthesis

## Abstract

Plants continuously acclimate to natural fluctuations in light intensity, and this may be modulated by elevated CO_2_ (e[CO_2_]) concentrations. How strongly such a combination affects plant and leaf morphology, anatomy, and photosynthetic biochemistry is unknown. We grew cucumber ( *Cucumis sativus* ) under sinusoidal (SN) and randomly fluctuating light (FL) intensities, at two CO_2_ levels (440 μmol mol^−1^, a[CO_2_]; 860 μmol mol^−1^, e[CO_2_]), and conducted an extensive analysis of photosynthesis, leaf anatomy, plant morphology, and biomass. Under a[CO_2_], plants demonstrated larger morphological and anatomical plasticity to FL than SN‐grown plants, with larger enhancements of stem height, leaf area, and specific leaf area (SLA). Surprisingly, leaf density instead of thickness explained the 70% higher SLA under FL than SN. Despite these changes, dry biomass was 25% lower under FL than SN, presumably due to lower photosynthetic efficiency and consequently lower diurnal photosynthesis under growth conditions (*A*_diurnal_). e[CO_2_] reduced the negative effect of FL on dry biomass, but was still 11% lower under FL compared to SN. This could be attributed to adjustments in SLA, enhanced shoot biomass, and *A*_diurnal_ under FL by e[CO_2_]. Overall, the negative impact of FL was partially mitigated under e[CO_2_] by adjustments in plant morphology and anatomy.

## 1 Introduction

In nature, light intensity fluctuates at timescales of milliseconds to hours. Such fluctuations are experienced by plants in the field and greenhouses due to self‐shading, passing clouds, and leaf movements induced by wind (Pearcy 1990; Way and Pearcy 2012; Burgess et al. 2021; Durand et al. 2021; van Westreenen et al. 2023), on the background of sinusoidally changing light intensity due to diurnal changes in solar angle. Besides fluctuating light (FL) and gradual sinusoidal (SN) changes in light intensity, greenhouse crops also experience elevated [CO_2_] (e[CO_2_]), as CO_2_ is often enriched in modern greenhouses to enhance growth (Poorter et al. 2022). This will also be the case for future field‐grown crops due to climate change, as [CO_2_] may reach 1100 μmol mol^−1^ by 2100 in the highest (SSP5‐8.5) emission scenario (IPCC 2023), with major repercussions for net photosynthesis rate (*A*), crop growth, and ecosystem functioning (Becklin et al. 2021). Despite the importance of both e[CO_2_] and FL, not much is known about plant acclimation to both factors, as their effects have been studied mostly separately. Acclimation to FL was often studied using a simple, square‐wave light intensity pattern with repeated and thus predictable fluctuations between high and low light intensities, which is not representative of natural light intensity patterns (Yin and Johnson 2000; Alter et al. 2012). Furthermore, studies on e[CO_2_] acclimation were typically carried out either on plants grown under steady growth light conditions or in free air [CO_2_] enrichment (FACE) studies, where other environmental factors also varied and [CO_2_] fluctuated strongly due to natural, frequent changes in wind direction and speed (Allen et al. 2020). To better understand acclimation to FL at different [CO_2_], there is thus a need to grow plants under FL patterns that more realistically represent stochastic fluctuations in nature (Vialet‐Chabrand et al. 2017) and to compare them to plants grown under sinusoidally changing light intensities at steady, well‐controlled CO_2_ conditions.

Plants respond to changes in their environment through several mechanisms which can be categorized as short‐term and long‐term responses; these occur at timescales ranging from seconds to minutes and from hours to days, respectively (Kono and Terashima 2014; Pearcy et al. 1996; Schöttler et al. 2015). Long‐term changes are acclimatory responses and are crucial for improving plant performance and fitness in the prevailing environment (Walters 2005; Athanasiou et al. 2010; Schumann et al. 2017). Photosynthetic acclimation involves adjustments in the composition of the photosynthetic apparatus that are not rapidly reversible (Walters 2005; Retkute et al. 2015). Alterations in gene expression often cause changes in protein abundance, which can translate to changes in tissue structure and composition. Acclimation can be categorized into two types: dynamic and developmental (Yin and Johnson 2000; Walters 2005; Athanasiou et al. 2010). Dynamic acclimation occurs in fully developed leaves through adjustment of existing structures and involves changes in pigment and protein composition (Athanasiou et al. 2010; Gjindali et al. 2021; Yin and Johnson 2000). Developmental acclimation occurs during leaf development and incurs changes in morphology, such as leaf thickness and stomatal density (Vialet‐Chabrand et al. 2017; Yin and Johnson 2000). In cucumber ( *Cucumis sativus* ), dynamic and developmental acclimation have been studied (Trouwborst et al. 2011; Pao et al. 2019; Yu et al. 2022), but not under FL. The fast developmental rate that cucumber plants show may result in specific architectural and morphological changes that impact acclimation responses.

Growth under FL often results in reduced biomass compared to constant light of the same average intensity (Blom and Zheng 2009; Vialet‐Chabrand et al. 2017; Morales and Kaiser 2020; Wei et al. 2021), although exceptions exist where no reduction was observed (Kaiser et al. 2018; Zhang et al. 2020; Bhuiyan and Van Iersel 2021). Growth under e[CO_2_] often increases biomass (Curtis and Wang 1998; Drag et al. 2020), but the strength of this effect depends on other environmental factors (Curtis and Wang 1998). As such, it can be expected that e[CO_2_] interacts with FL. Under FL, photosynthetic efficiency can be reduced due to at least three short‐term mechanisms: (i) a slow induction of photosynthesis after sudden increases in light intensity due to slow activation of Calvin cycle enzymes and stomatal opening, (ii) slow deactivation of non‐photochemical quenching (NPQ) after transitions from high to low light intensity, and (iii) low photosynthetic quantum yield at high light intensity (Morales and Kaiser 2020). In the longer term, photoinhibition can also play a role by reducing photosynthesis efficiency depending on the intensity reached during the light fluctuations (Burgess et al. 2015). The low photosynthetic efficiency observed under FL could be mitigated by e[CO_2_]. However, the combined acclimation response to FL and e[CO_2_] could lead to opposite or combined responses. Leaf photosynthesis efficiency under FL has been shown to be influenced by changes in leaf morphology and anatomy (Vialet‐Chabrand et al. 2017; Matthews et al. 2018). As such, the effect of FL and e[CO_2_] on leaf morphology and anatomy can also influence the combined acclimation response.

Plasticity of leaf anatomy can be analyzed relative to specific leaf area (SLA; Poorter et al. 2022). High SLA results in thinner and relatively larger leaves and is thought to enhance light interception per unit mass (Liu et al. 2016). Acclimation to FL often increased SLA (Morales and Kaiser 2020; Vialet‐Chabrand et al. 2017; Wei et al. 2021; Zhang et al. 2020), whereas acclimation to e[CO_2_] decreased SLA (Ainsworth and Long 2005; Leakey et al. 2002; Mizokami et al. 2019; Poorter et al. 2022; Ruiz‐vera et al. 2021). Changes in SLA can be driven by changes in leaf thickness, leaf density, or a combination of both (Poorter et al. 2009). A larger SLA under FL was previously found to be due to reduced leaf thickness, which was caused by a reduction of both the palisade and the spongy cell layers (Vialet‐Chabrand et al. 2017; Zhang et al. 2020), and opposite effects were observed under e[CO_2_] (Zheng et al. 2019). Conversely, Mizokami et al. (2019) noticed a reduction in SLA in *Arabidopsis thaliana* under e[CO_2_] that was caused by an increase in leaf density, which was driven by thicker cell walls and starch accumulation, but not by a change in leaf thickness. Such changes in SLA in response to environmental factors can affect photosynthesis by altering light distribution and CO_2_ diffusion in the leaf (Ren et al. 2019). Further, given the contrasting effects of FL and e[CO_2_] on SLA, investigating their combined effects on leaf traits is important. In addition to these anatomical adjustments, vein density acclimated particularly in response to light (sun and shade conditions; Brodribb and Jordan 2011; Carins Murphy et al. 2012), but not to e[CO_2_] (Uhl and Mosbrugger 1999). e[CO_2_] acclimation typically has only slight negative, positive, or no effects on stomatal density (Ainsworth and Rogers 2007; Poorter et al. 2022; Wall et al. 2023). Acclimation to FL either caused a reduction or no change in stomatal density (Matthews et al. 2018; Zhang et al. 2020; Wei et al. 2021). Stomatal and vein density often respond coordinatively and vein density is often positively correlated with hydraulic conductance and leaf gas exchange (Brodribb and Jordan 2011; Carins Murphy et al. 2012; Ye et al. 2021). Upon acclimation to FL, reductions in photosynthetic capacity, electron transport efficiency of PSII (*ɸ*_PSII_), chlorophyll content, chlorophyll *a*:*b* ratio, and photosynthetic protein contents were observed (Vialet‐Chabrand et al. 2017; Wei et al. 2021). Similarly, acclimation to e[CO_2_] generally reduces photosynthetic capacity and protein contents (Ainsworth and Long 2005; Ainsworth and Rogers 2007; Chen et al. 2005; Tomimatsu et al. 2019; Zheng et al. 2019). Overall, acclimation to a combination of FL and e[CO_2_] may change leaf anatomical and morphological features such that the negative effects of FL on growth are alleviated.

Little is known about the combined long‐term effects of e[CO_2_] and FL on plants. To our knowledge, the only study on the topic found that e[CO_2_] but not FL caused photosynthetic acclimation (Leakey et al. 2002). However, this study was conducted on the tropical rainforest tree species *Shorea leprosula* (Leakey et al. 2002), which may respond to these treatments very differently than fast‐growing crop species. Therefore, we aimed to investigate how e[CO_2_] and FL affect plant growth and leaf morphology, anatomy, and photosynthesis in cucumber, an economically important horticultural crop that has repeatedly been used to study light acclimation (Chen et al. 2018; Pao et al. 2019). The FL treatment was compared to a slowly changing SN light intensity (control) treatment, which is closer to natural conditions than square wave light intensity treatments. Furthermore, we also aimed to address whether acclimation to the combination of FL and e[CO_2_] can mitigate the negative effects of FL acclimation on plant growth and photosynthesis and if this happens predominantly due to morphological, anatomical, and/or biochemical adjustments. We hypothesized that: (i) compared to slowly changing SN light, growth under FL will negatively impact *A* and plant growth, (ii) e[CO_2_] will reduce the negative impacts of FL on photosynthesis and plant growth, (iii) at a[CO_2_], FL‐grown plants will have lower photosynthetic capacity, stomatal and vein densities, compared to control treatment, as well as higher SLA, but that at e[CO_2_] the effects of FL on these traits would be canceled out.

## 2 Materials and Methods

### 2.1 Plant Material and Growth Conditions

Plants were grown in a climate room at Wageningen University and Research, the Netherlands. Cucumber ( *Cucumis sativus* cv. Hi‐Power, Nunhems (BASF), Nunhem, the Netherlands) seeds were sown in rockwool plugs (2 cm diameter; Grodan, Roermond, the Netherlands). After 7 days, seedlings were transplanted to rockwool blocks (10 × 10 × 6.5 cm; Grodan) and grown for 29 days under their respective treatments. Side shoots were removed when they were < 2 cm to maintain monopodial growth. Plants were irrigated twice a day with nutrient solution (pH 5.8, EC 2 dS m^−1^) using an automatic ebb and flood system. The nutrient solution was composed of NH_4_^+^ 1.2 mM L^−1^, K^+^ 7.2 mM L^−1^, Ca^2+^ 4 mM L^−1^, Mg^2+^ 1.82 mM L^−1^, NO_3_^−^ 12.4 mM L^−1^, SO_4_^2−^ 3.32 mM L^−1^, H_2_PO_4_^−1^.1 mM L^−1^, Mn 10 μM L^−1^, Zn 5 μM L^−1^, B 30 μM L^−1^, Cu 0.75 μM L^−1^, Mo 0.5 μM L^−1^, and Fe 25 μM L^−1^. Setpoints for growth conditions were 23°C/20°C day/night temperature, 70% relative humidity (RH), and 16 h photoperiod. Light was provided by dimmable LED lamps (GreenPower LED Toplighting; Signify, Eindhoven, the Netherlands) with a spectrum of 6% blue (400–500 nm), 10% green (500–600 nm), 84% red (600–700 nm), and 10% far red (700–800 nm).

Plant positions on the growth table were randomized every 3 days to reduce position effects on plant growth and development. Plant spacing was adjusted regularly such that plants did not shade each other. The youngest fully mature leaf was selected as leaf #6, counted from the bottom, with the first true leaf being leaf #1. Three to five days before the start of gas exchange measurements (described below), leaf #6 was fixed horizontally (Figure S1) using a customized leaf support structure to ensure that all measurement leaves of different treatments were acclimated to the same average light intensities and to their respective light patterns.

### 2.2 Light Patterns and [CO_2_ ] Treatment Combinations

Cucumber seedlings were subjected to four different combinations of light patterns (fluctuating and SN light) and [CO_2_] levels (440 μmol mol^−1^ [aCO_2_] and 860 [eCO_2_] μmol mol^−1^): (1) FL × aCO_2_, (2) FL × eCO_2_, (3) SN × aCO_2_, and (4) SN × eCO_2_. The SN and FL light patterns were chosen as they mimic cloudless above‐canopy and windy in‐canopy (intermittent self‐shading) situations, respectively. A programmable controller was used to control the light intensities. SN light treatment followed a SN pattern that peaked at ca. 320 μmol m^−2^ s^−1^ at noon (Figure S2). For the FL treatment, light intensity fluctuated randomly between 120 and 1200 μmol m^−2^ s^−1^ (at table height) every 1 and 5 min, and it also followed a SN pattern. Although the overall shape, duration, and average light intensity were kept identical, the exact pattern of FL was randomized daily, resulting in day‐to‐day variation of several characteristics: there were 289 ± 30 light intensity shifts (average ± standard deviation; *n* = 120 days) per photoperiod in the FL treatment (Table S3). The duration at the lowest light intensity during the photoperiod, 120 μmol m^−2^ s^−1^, was 9.7 h, the duration of PPFD between 121 and 400 μmol m^−2^ s^−1^ was 3.6 h, and the duration of PPFD > 400 μmol m^−2^ s^−1^ was 2.7 h (Figure S3 and Table S3). The largest PAR amplitude per photoperiod was 1074 μmol m^−2^ s^−1^ (Table S3). Both light intensity patterns had an average light intensity (400–700 nm) of 250 μmol m^−2^ s^−1^ and a photoperiod of 16 h (daily light integral: 14.4 mol m^−2^ d^−1^). Each combination of treatment was conducted twice through time, with three individual plants measured per treatment. In total, six plants were measured per treatment.

### 2.3 Measurements of Leaf Traits

All measurements were conducted on leaf #6, after 27–29 days of treatment. Non‐steady state gas exchange and combined gas exchange and chlorophyll fluorescence measurements were conducted on the same plant on the 27th and 28th day, respectively. On the 29th day, stomatal imprints were taken and a destructive harvest was conducted. Leaf discs were taken for vein density, leaf cross‐sections, and leaf optical property analysis. The rest of leaf #6 was oven‐dried for SLA calculation. Diurnal gas‐exchange measurements were conducted on another set of plants on the 29th day of treatment. Leaves number #6 of another set of plants were frozen at 11.00 for leaf pigment and carbohydrate analysis.

#### 2.3.1 Combined Gas Exchange and Chlorophyll Fluorescence

Photosynthetic gas exchange and chlorophyll fluorescence (CF) measurements were conducted on leaf #6 using a portable gas exchange system (LICOR‐6800, Li‐Cor Bioscience) equipped with a 6800‐01A fluorometer (enclosed leaf area: 2 cm^2^). Unless stated otherwise, measurements were performed at a RH of 70%, air temperature of 25°C, flow rate of 300 μmol s^−1^, and [CO_2_] of 400 μmol mol^−1^. Light was provided by a mixture of red (90%) and blue (10%) LEDs in the fluorometer. Peak wavelengths of red and blue LEDs in the fluorometer were 625 nm and 475 nm, respectively.

##### 2.3.1.1 *A* /PPFD and *A* / *C*_*i*_ Curves of Cucumber Leaves

Leaves were dark adapted in the LI‐6800 cuvette for 30 min, after which minimal (*F*_0_) and maximal (*F*_*m*_) fluorescence was measured (at ca. 11.00 in the morning). Light intensity (photosynthetic photon flux density, PPFD) was then increased to 250 μmol m^−2^ s^−1^, and gas exchange was logged every 5 s for 30 min. Then, PPFD was increased to 2000 μmol m^−2^ s^−1^, and gas exchange was logged for at least 5 min every 5 s until the stability criterion (slope of change in *A*: < 0.05 μmol m^−2^ s^−1^ for > 20 s) was met or for a maximum wait time of 20 min. Then, PPFD was decreased in steps of 1500, 1000, 800, 600, 400, 200, 150, 100, 50, and 0 μmol m^−2^ s^−1^ with a minimum wait time of 60 s. Gas exchange, *F*_*m*_′, and *F*′ were logged either when stability was met or when the maximum wait time of 180 s was reached. Then, leaves were re‐exposed to 1500 μmol m^−2^ s^−1^ for at most 20 min, and *A* was measured at [CO_2_] steps of 400, 300, 200, 100, and 50 μmol mol^−1^. At each step, the minimum wait time was 120 s, and gas exchange and CF were logged when the stability criterion was met or when the maximum wait time of 300 s had been reached. Then, leaves were adapted to 400 μmol mol^−1^ [CO_2_] for a minimum wait time of 5 min, and gas exchange and CF were logged when either stability was met or when a maximum wait time of 20 min was reached. Then, [CO_2_] was increased in steps of 600, 900, 1200, 1500, and 1800 μmol mol^−1^ with a minimum wait time of 60 s. Gas exchange and CF were logged when either stability was met or when a maximum wait time of 5 min was reached. CO_2_ and H_2_O were matched at each step before the data was logged.

At each light and [CO_2_] step, a rectangular saturating flash was applied to determine CF under actinic light (*F*_*s*_) and maximum CF (*F*_*m*_′). A saturating flash of 13,000 μmol m^−2^ s^−1^ was applied for 300 ms, at a measuring beam frequency of 100 Hz. These settings had been determined to be optimal in a preliminary experiment.

##### 2.3.1.2 Non‐Steady State Gas Exchange of Cucumber Leaves

For assessing the response of *A* and *g*_*s*_ to step changes in PPFD, leaves were first dark‐adapted for 20 min. Then, PPFD was increased to 120 μmol m^−2^ s^−1^ until *A* and *g*_*s*_ were stable (~70 min). Then, PPFD was increased to 1200 μmol m^−2^ s^−1^ for 60 min, after which it was decreased to 120 μmol m^−2^ s^−1^ for 90 min. *A* and *g*_*s*_ were logged every 2 s throughout.

##### 2.3.1.3 Diurnal Gas Exchange of Cucumber Leaves

Diurnal gas exchange measurements were conducted for the whole day on the 29th day of the treatment. For diurnal measurements of photosynthetic gas exchange, a program of 16 h duration was set that tracked the PPFD in the climate chamber continuously using the Li‐190 PAR sensor belonging to the fluorometer. Other conditions were 65% RH, 23°C temperature, and 440 or 860 μmol mol^−1^ [CO_2_], depending on CO_2_ treatment. Gas exchange was logged every 5 s, and CF was measured every 5 min. The operational respiration rate (*R*_op_) was calculated by taking an average (over a period of 4 min) of *A*, 13 min after the lights were switched off. Diurnal quantum yield of CO_2_ assimilation (*ɸ*_CO2_) was calculated by dividing gross *A* (i.e., *A* + *R*_op_) by absorbed PPFD. *A*_diurnal_ was calculated by integrating diurnal *A* between 9.00 and 23.00; this period was shortened from initially 16 to 14 h to avoid that minor inconsistencies in timing between replicates affected the integrated number.

#### 2.3.2 Stomatal Morphology of Cucumber Leaves

To create an imprint of the stomata, Zhermack elite HD+ silicon (Zhermack SpA, Badia Polesine) was dotted onto leaf #6. Per leaf, four imprints were taken on the bottom leaf surface and another four on the top surface. Clear nail polish was applied to the impression, peeled off when dry, and viewed under the microscope (Leitz Aristoplan; Leica Microsystems). Pictures were taken at 25× and 40× objective (camera: Axiocam 305 color; Carl Zeiss) and analyzed using the ObjectJ and CellCounter plugins in ImageJ v1.54f (National Institute of Health). Stomatal density, size, and index were calculated following Savvides et al. (2012).

#### 2.3.3 Destructive Harvest of Cucumber Plants

Plants were harvested 29 days after the start of treatments. Leaf area was measured using a leaf area meter (Li‐3100C; Li‐Cor Biosciences). Fresh weights of whole shoot biomass as well as that of leaves, stem, petioles, fruits, and flowers were determined. Organs were dried at 70°C for 3 days in the oven, after which their dry weights were determined.

#### 2.3.4 Leaf Optical Properties

Leaf light reflectance and transmittance were measured on leaf discs using two integrating spheres (Hogewoning et al. 2010). Leaf light absorptance was calculated as 1 − reflectance − transmittance.

#### 2.3.5 Leaf Chlorophyll and Carotenoid Levels

For leaf pigment analysis, 1.5 mL of 80% acetone buffered with 25‐mM HEPES of pH 7.5–7.8 was added to 10 mg of freeze‐dried ground leaf sample and vortexed. It was then left in the dark at 4°C for 10 min and centrifuged at 4°C for 10 min at 10,000 g (Centrifuge 5425R; Eppendorf). The supernatant was diluted four times and measured using the plate reader (SpectraMax iD3, Molecular Devices). The absorbance was determined at 470, 647, 664, and 750 nm. Chlorophyll concentrations were calculated based on Porra et al. (1989), and carotenoids based on Lichtenthaler (1987).

#### 2.3.6 Leaf Vein Density

Leaf discs of 2 cm^2^, sampled between secondary veins, were put in 5% NaOH for 20–24 h. Discs were washed with water three times, kept in water overnight, and bleached using 50% NaClO until they turned white (about 30–60 min). A dehydration series of 40%, 60%, 80%, and 96% ethanol was used for 5 min per ethanol step. Discs were stained with 1% safranin for 1 h, and then destained in an ethanol series of 96%, 80%, 60%, and 40% for 20 min each, until excess stains on the discs were removed. Discs were viewed under the stereo microscope (Leica MZ APO, Leica Microsystems), and six pictures were taken at a magnification of 3.2× (Axiocam 305 color). For analyzing vein pictures (Figure S4), ImageJ v1.54f was used. Lines (blue colored, 15 μm) were manually drawn over the veins. Then, the color channel was split and blue was retained for further analysis. Pictures were thresholded and skeletonized (using the skeletonize plugin in ImageJ). The total number of pixels of the skeleton was counted and divided by the total number of pixels of the image to obtain vein density (no. per μm^2^).

#### 2.3.7 Leaf Cross Sections

Leaf discs were fully immersed in FAA (37% formaldehyde: acetic acid: 70% ethanol; volume ratios of 5:5:90). A dehydration series with 70%, 85%, 96%, and 100% ethanol was carried out for 2 h at each ethanol step and then stored overnight in 100% ethanol. Then, leaf discs were kept in a series of Technovit solution (1 g hardener I in 100 mL Technovit 7100; Kulzer GmbH) of 50% (with 100% ethanol) and 100% concentration for 2 h each and then stored at 4°C. Discs were positioned in plastic molds by adding Technovit 7100 solution mixed with hardener II and kept at room temperature for 1 h and then in an oven at 40°C for 4 h. Samples were cut using a microtome (Reichert–Jung 2050; R. JUNG GmbH) into 5 μm thick slices and placed on slides. Samples were stained using 1% toluidine blue for 5 min and washed a few times with water to remove excess stains. The slides were then viewed under the microscope (Leitz Aristoplan, with a 10× eyepiece) with a 25× objective and photographed using the Axiocam 305 camera. Ten to twelve pictures (Figure S5) per leaf were measured using ImageJ, and the lengths of the overall cross‐section, palisade, and spongy mesophyll were measured.

#### 2.3.8 Leaf Carbohydrate Contents

Carbohydrates were measured according to Larsen et al. (2022): 15 mg of freeze‐dried, ground leaf samples were mixed with 5 mL 80% ethanol, vortexed, and kept in a shaking water bath at 80°C for 20 min. Then, samples were centrifuged for 5 min at 8500 g (UNIVERSAL 320R Centrifuge, Hettich Zentrifugen). One milliliter supernatant was dried in a vacuum centrifuge (Savant SpeedVac SPD2010; Thermo Fisher Scientific) at 45°C and 5.1 mbar for 140 min. The remaining supernatant with pellet was stored at −20°C for starch determination. Dried samples were resuspended in 2 mL 0.01 N HCl and put in an ultrasonic water bath (BRANSON 2800 Ultrasonic Cleaner; Branson Ultrasonics) for 10 min. Then, samples were vortexed and centrifuged at 4°C for 5 min at 9391 g (Centrifuge 5425R; Eppendorf). To remove excess amino acid compounds, samples were filtered using solid phase extraction (SPE) columns (Clean‐Up BCX SPE Cartridges, 100 mg, 1 mL [UCT]), rinsed with 3 mL Milli‐Q water and 5 mL of 0.01 N HCl. Samples were diluted 10 times and concentrations of glucose, fructose, sucrose, and stachyose were measured with a high‐performance ion chromatograph (ICS‐5000, Thermo Fisher Scientific) with a CarboPac PA1 (2 × 250 mm) column (Thermo Fisher Scientific) at 25°C with 100‐mM NaOH as eluent at the flow rate of 0.25 mL min^−1^. Chromeleon 7 (Thermo Fisher Scientific) was used for the analysis of the chromatograms.

The remaining pellet was washed three times with 80% ethanol and dried in a vacuum centrifuge for 40 min (Savant SpeedVac SPD2010, Thermo Fisher) at 45°C and 5.1 mbar. Two milliliters of 1 mg ml^−1^ alpha‐amylase (SERVA Electrophoresis GmbH) was added and put into a shaking water bath at 90°C for 30 min. To this mixture, 1 mL of amyloglucosidase (10115 Sigma–Aldrich; 0.5 mg ml^−1^ in 50 mM citrate buffer, pH: 4.6) was added and again put into the shaking water bath at 60°C for 15 min. The mixture was centrifuged at 8500 g for 5 min and diluted 50 times. Diluted samples were then measured with the high‐performance ion chromatograph as described above.

#### 2.3.9 Leaf Carbon and Nitrogen Contents

For the carbon and nitrogen analysis, 150 mg oven‐dried, ground samples were used and measured using the LECO FP828 combustion nitrogen/protein determinator, using Argon (10 cc) as the carrier gas (LECO Corporation). The carbon and nitrogen content per unit mass was converted to an area basis by multiplying by leaf mass area (LMA; leaf dry weight divided by leaf area).

### 2.4 Calculations

#### 2.4.1 *A* /PPFD Curve

A non‐rectangular hyperbola was fit to the *A*/PPFD data based on Lobo et al. (2013).

#### 2.4.2 *A* / *C*_*i*_ Curve

The parameters *V*_*c*max_ and *J* were determined by fitting the data using the Sharkey (2016) tool. All *C*_*i*_ values below 220 μmol mol^−1^ were assigned to Rubisco limitation, those above 220 μmol mol^−1^ to RuBP limited, and points where *A* did not increase with increasing *C*_*i*_ were assigned to TPU limited. For the sake of A/*C*_*i*_ curve fitting, mesophyll conductance was assumed to be 0.3 mol m^−2^ s^−1^, based on Moualeu‐Ngangue et al. (2017). To convert *A* per unit leaf area to *A* per unit mass (*A*_mass_), *A* was divided by LMA.

#### 2.4.3 *A* Induction

The photosynthetic induction state (IS) after a step increase in light was calculated as:

$$\text{IS}\left(t\right)=\frac{A\left(t\right)-A_{i}}{A_{f}-A_{i}}$$

where *A*(*t*) (μmol m^−2^ s^−1^) is the rate of photosynthesis at time *t*; *A*_*i*_ and *A*_*f*_ are the steady‐state *A* at low and high light, respectively. The IS at 60 s (IS_60_ [%]), time (s) to reach 50 (*T*_50_) and 90% of IS (*T*_90_) were calculated.

#### 2.4.4 Temporal *g*_*s*_ Response

The *g*_*s*_ response to step increase and decrease in light was assessed using the following equation:

$$g_{s}=g_{i}+\left(g_{f}-g_{i}\right)\left(1-e^{-{\left(\frac{t}{\tau }\right)}^{\lambda }}\right)$$

where *g*_*i*_ is the initial steady‐state *g*_*s*_ at low light; *g*_*f*_ is the steady‐state *g*_*s*_ at high light, *τ* is the time constant (s) that represents the time to reach 63% of change in *g*_*s*_ over *t*; *λ* is the shape constant that determines the shape of the curve.

### 2.5 Statistical Analysis

The experiment was conducted twice. In each experiment, there were three replicate plants per treatment. All statistical analyses were performed using GenStat 22nd edition (VSN International Ltd.). The assumptions for normality and homogeneity were tested using Shapiro‐Wilk's test and Bartlett's test, respectively. Two‐way analysis of variance (ANOVA) with interaction between light pattern and CO_2_ level was performed at *p* < 0.1. Due to the limited number of replicate experiments, the significant difference between the treatments was assessed at *p* < 0.1 as in Kaiser et al. (2019). If the interaction effect was significant, Fisher's protected LSD test was performed at *p* < 0.1. Effects of light and [CO_2_] treatments are mostly displayed as interaction plots, with [CO_2_] on the *x*‐axis.

## 3 Results

### 3.1 Plant Growth and Morphology

**FIGURE 1:**
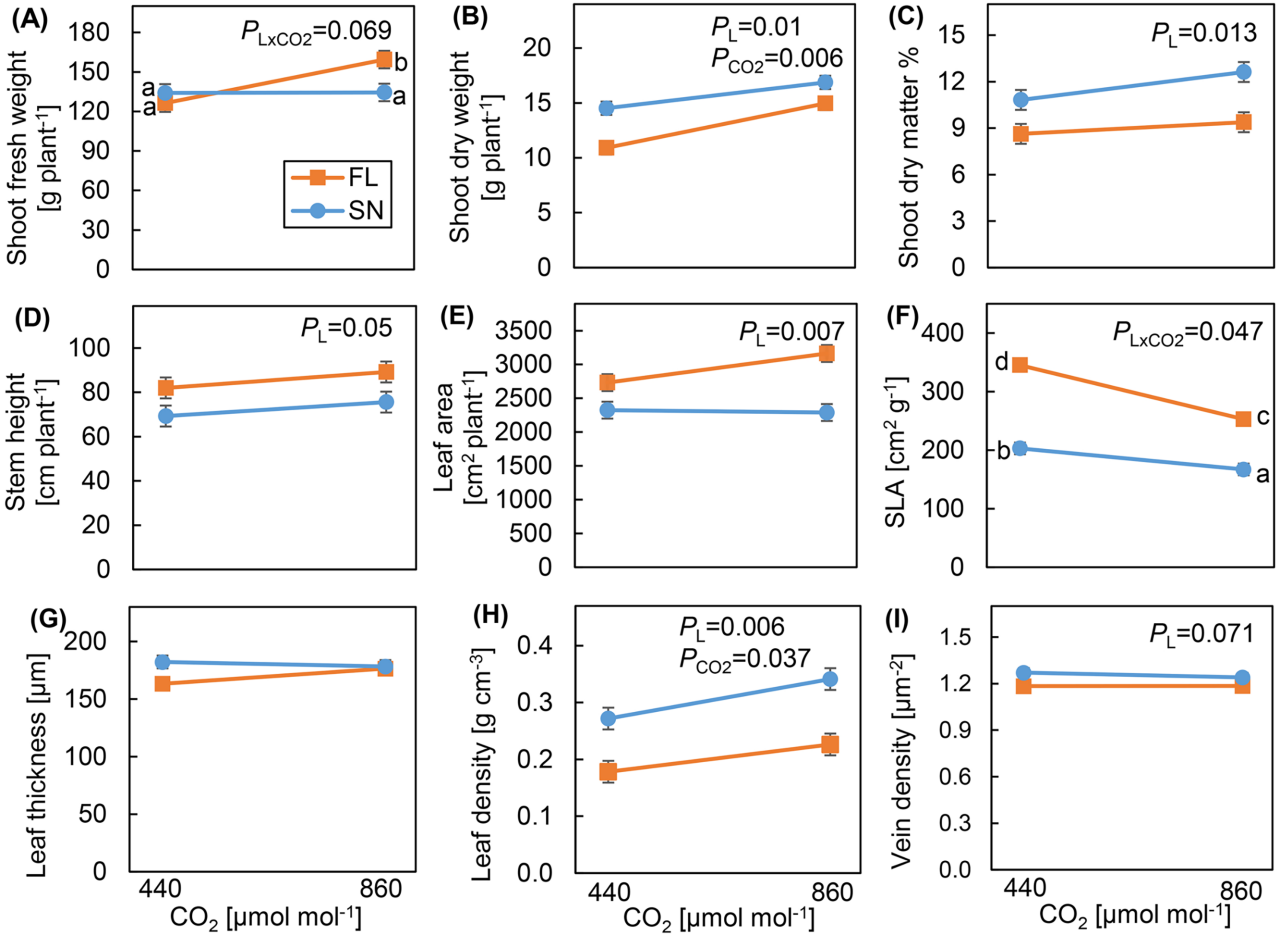
Growth, leaf morphology, and anatomy of cucumber plants grown under different light patterns and [CO_2_]. (A) shoot fresh weight, (B) shoot dry weight, (C) shoot dry matter percentage, (D) stem height, (E) leaf area, (F) specific leaf area (SLA, ratio of leaf area to leaf dry weight), (G) leaf thickness, (H) leaf density (ratio of 1/SLA to leaf thickness), and (I) vein density. Cucumber plants were grown under four treatments: SN × aCO_2_ (sinusoidal light + ambient CO_2_), SN × eCO_2_ (sinusoidal light + elevated CO_2_), FL × aCO_2_ (fluctuating light + ambient CO_2_), and FL × eCO_2_ (fluctuating light + elevated CO_2_). Data represent means ± SEM based on two experiments each with three replicate plants per experiment. *p* values of the main effects of light pattern (*P*_*L*_), CO_2_ (*P*_CO2_), and their interaction (*P*_*L*×CO2_) are shown, when *p* < 0.1. Different letters indicate significant differences between treatments.

Light pattern and [CO_2_] had strong effects on above‐ground biomass, and affected related traits interactively (Figure 1). Under e[CO_2_], FL‐grown plants had substantially higher fresh mass (19%) than SN‐grown plants, while under a[CO_2_], no significant difference was observed between light treatments (Figure 1A). A different pattern was observed for dry mass, which was reduced in FL‐grown plants under both [CO_2_], and more strongly so under a[CO_2_] (−25%) than under e[CO_2_] (−11%; Figure 1B). The differences observed between fresh and dry mass (Figure 1A,B) were explained by substantially lower dry matter percentage in FL‐grown plants than that in SN‐grown plants (Figure 1C). Additionally, there was a nonsignificant tendency for e[CO_2_] grown plants to increase dry matter percentage compared to a[CO_2_] grown plants (Figure 1C). Stems of FL‐grown plants were significantly taller in both CO_2_ treatments than those of SN‐grown plants (Figure 1D). Moreover, FL‐grown plants had 28% larger leaf area than SN‐grown plants at both CO_2_ treatments (Figure 1E). e[CO_2_] tended to increase leaf area in FL plants compared to a[CO_2_], but there was no such trend in SN‐grown plants (*p* = 0.136; Figure 1E). In general, the highest growth was observed in plants grown under e[CO_2_] and SN.

**FIGURE 2:**
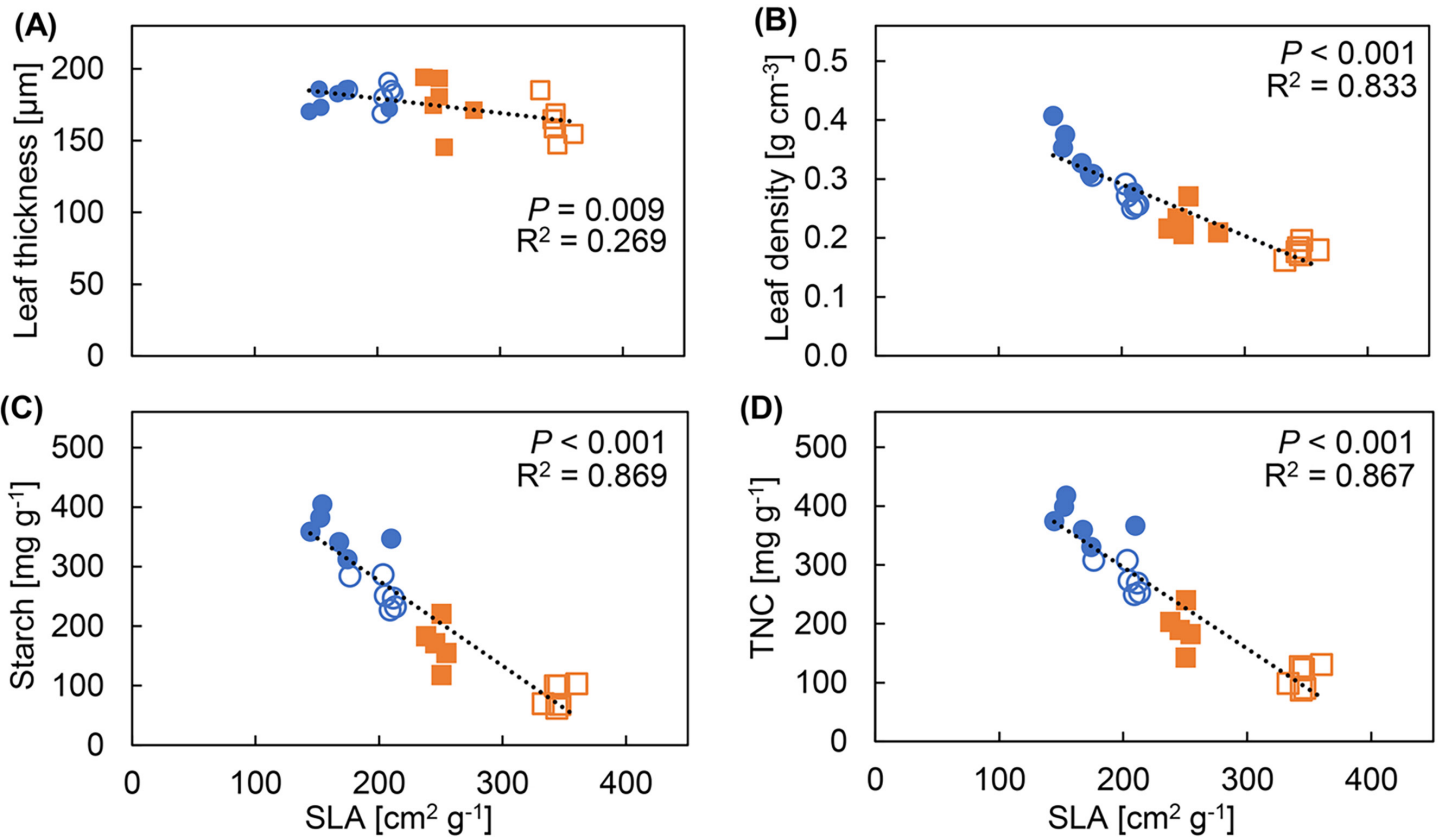
Correlation analysis of SLA with leaf anatomical traits and carbohydrate content. Correlations of SLA with (A) leaf thickness, (B) leaf density, (C) starch, and (D) total non‐structural carbohydrates (TNC; sum of glucose, fructose, sucrose, stachyose, and starch). Cucumber plants were grown under four treatments: SN × aCO_2_ (sinusoidal light + ambient CO_2_; clear circles), SN × eCO_2_ (sinusoidal light + elevated CO_2_; filled circles), FL × aCO_2_ (fluctuating light + ambient CO_2_; clear squares), and FL × eCO_2_ (fluctuating light + elevated CO_2_; filled squares). Pearson's correlation was performed on single replicate values with *n* = 6 replicate plants per treatment.

The larger leaf area in FL‐grown plants was accompanied by a much higher SLA at both CO_2_ concentrations than SN‐grown plants: especially under a[CO_2_], SLA was increased by ~70% in FL, whereas at e[CO_2_] that difference was still ~50% (Figure 1F). Surprisingly, despite these large differences in SLA, we did not observe any treatment effects on leaf thickness (Figure 1G) nor on palisade mesophyll thickness (Figure S6A). Although FL‐grown plants showed a reduced length of the spongy mesophyll, this difference was relatively minor (Figure S6B). Also, FL‐grown plants had significantly lower vein density than SN‐grown plants, but this difference was only ~6% (Figure 1I). In an attempt to explain the changes in SLA that occurred without changes in leaf thickness, we calculated leaf density as the ratio between leaf mass area and leaf thickness: This analysis suggested that FL‐grown leaves had much lower leaf density than SN‐grown leaves, and leaves grown under e[CO_2_] had significantly higher leaf density than those under a[CO_2_] (Figure 1H). Furthermore, despite significant correlations of SLA with leaf thickness and leaf density, the magnitude of correlation was greater with leaf density than with leaf thickness (Figure 2A,B). Also, SLA correlated negatively with both starch and total non‐structural carbohydrates (TNCs; Figure 2C,D). Leaf density also correlated positively with starch content (*R* ^2^ = 0.92; *p* < 0.001).

### 3.2 Effect of Light Patterns and [CO_2_ ] Acclimation on Photosynthesis

**FIGURE 3:**
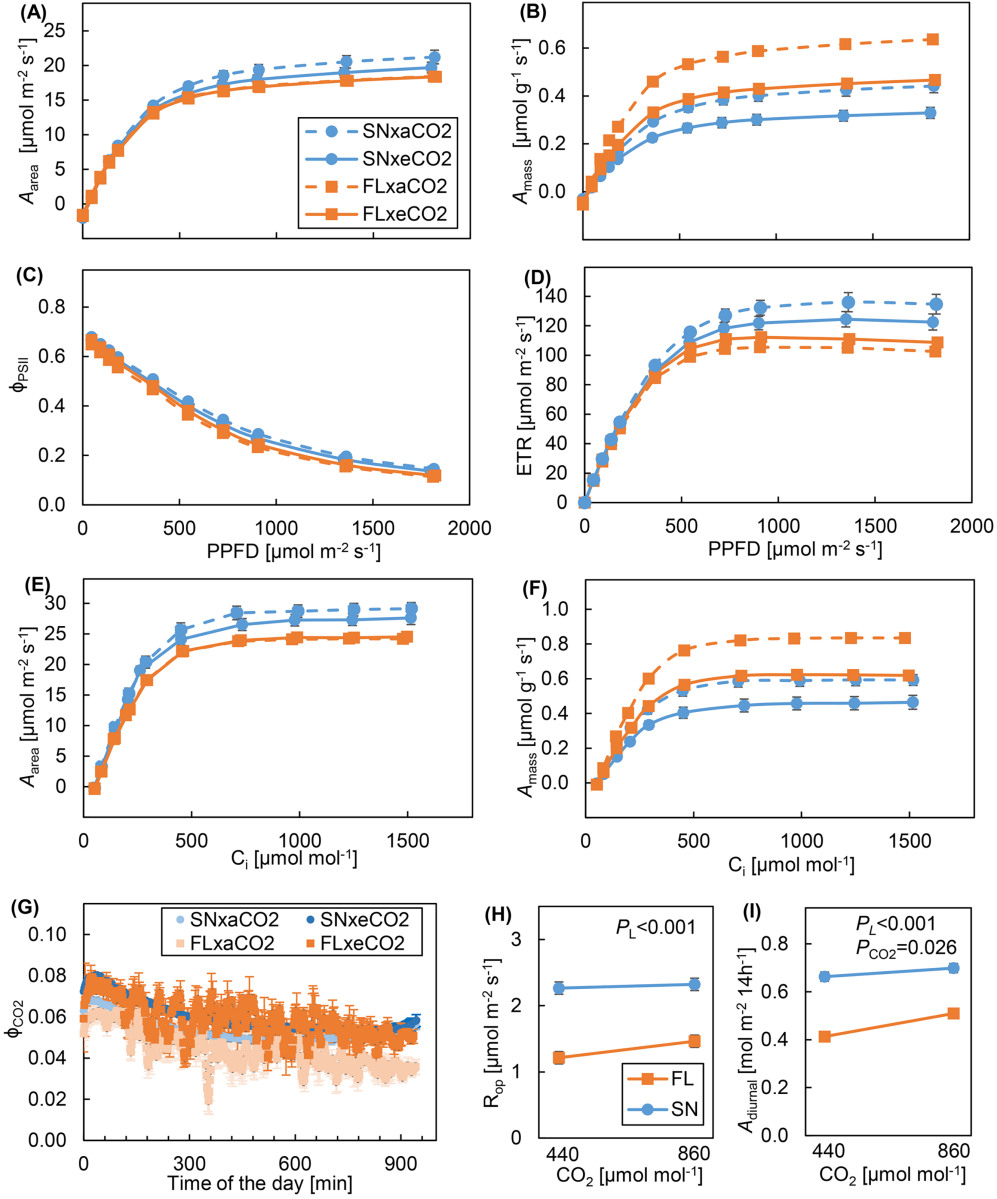
Steady‐state and diurnal photosynthesis of cucumber leaves grown under different light patterns and [CO_2_]. Steady‐state photosynthesis as a function of light absorbed by the leaf (PPFD): (A) net photosynthesis rate per unit leaf area (*A*_area_), (B) photosynthesis rate per unit mass (*A*_mass_), (C) PSII operating efficiency (*ɸ*_PSII_), and (D) electron transport rate (ETR). Steady‐state photosynthesis as a function of intercellular CO_2_ concentration (*C*_*i*_): (E) *A*_area_, and (F) *A*_mass_. Diurnal photosynthesis measurements at the respective growth conditions: (G) *ɸ*_CO2_ during the photoperiod, (H) operating respiration rate (*R*_op_), and (I) integrated diurnal photosynthesis for 14 h (*A*_diurnal_). Cucumber plants were grown under four treatments: SN × aCO_2_ (sinusoidal light + ambient CO_2_), SN × eCO_2_ (sinusoidal light + elevated CO_2_), FL × aCO_2_ (fluctuating light + ambient CO_2_), and FL × eCO_2_ (fluctuating light + elevated CO_2_). Means ± SEM of 5–6 plants are shown for the steady‐state photosynthesis and diurnal photosynthesis measurement. Data represent means ± SEM based on two experiments each with 2–3 replicate plants per experiment for *R*_op_ and *A*_diurnal_. *p* value of the main effect of light pattern (*P*_*L*_) and CO_2_ (*P*_CO2_) is shown, when *p* < 0.1.

In FL‐grown plants, *A* at high PPFD tended to be lower than that in SN‐grown plants (Figure 3A), although no significant difference was found in the derived parameters (*A*_sat_, *R*_*d*_, *α*, *θ*; Table 1). Similarly, at higher *C*_*i*_, FL‐grown plants had lower *A* and *A*_max_ compared with SN‐grown plants (Figure 3E and Table 1), with a reduced TPU and a nonsignificant tendency for lower *V*_*c*max_ and *J* (Table 1). To account for the large differences in SLA between treatments (Figure 1F), we calculated *A* per unit leaf mass (*A*_mass_). In both *A*/PPFD and *A*/*C*_*i*_ curves, *A*_mass_ in FL‐grown leaves was much larger than in SN, and was further enhanced in a[CO_2_] compared to e[CO_2_] (Figure 3B,F); these differences showed in all derived parameters (Table 1). Also, when calculating leaf dry mass minus TNCs and expressing *A*_mass_ on that basis, *A*_mass_ was still higher in FL than SN‐grown plants (Figure S7G,H). The maximum *ɸ*_PSII_ (*F*_*v*_/*F*_*m*_) was similar between treatments (Figure S7D). PSII operating efficiency (*ɸ*_PSII_) tended to be somewhat lower in FL‐grown plants along the *ɸ*_PSII_/PPFD curve compared to SN‐grown plants, but this was not significant (Figure 3C). A similar pattern was observed for the PSII efficiency factor (qp; Figure S7B). Similarly to the *A*/PPFD curve, the electron transport rate was lower for FL‐grown plants > 400 μmol m^−2^ s^−1^ PPFD compared to SN‐grown plants (Figure 3D). At PPFD < 400 μmol m^−2^ s^−1^, while maximum PSII efficiency (*F*_*v*_′/*F*_*m*_′) tended to be lower, NPQ tended to be higher in FL‐grown plants, while at higher light intensities, no treatment effect was observed (Figure S7A,C).

**TABLE 1:** Steady‐state photosynthesis parameters used in this work: Maximum photosynthesis rate at saturating light (*A*_sat_), mitochondrial respiration from *A/*PPFD curve (*R*_*d*_), quantum yield (*α*), convexity parameter (*θ*), maximum photosynthesis rate at saturating light and CO_2_ (*A*_max_), maximum carboxylation rate (*V*_*c*max_), electron transport rate (*J*), and triose phosphate utilization (TPU).

Parameters	Treatment				*p*		
	SN × aCO_2_	SN × eCO_2_	FL × aCO_2_	FL × eCO_2_	*L*	CO_2_	*L* × CO_2_
*A*_sat_ (μmol m^−2^ s^−1^)	23.2	21.0	19.3	19.5	0.108	0.479	0.429
*R*_*d*_ (μmol m^−2^ s^−1^)	2.0	1.9	1.6	1.9	0.261	0.744	0.282
*α* (μmol mol^−1^)	0.076	0.075	0.070	0.071	0.236	0.921	0.892
*θ*	0.70	0.67	0.74	0.74	0.152	0.563	0.662
*A*_sat_mass_ (μmol g^−1^ s^−1^)	0.47	0.35	0.67	0.49	**0.012**	**0.017**	0.513
*R*_*d*_mass_ (μmol g^−1^ s^−1^)	0.041	0.031	0.054	0.047	**0.036**	0.126	0.822
*α*_mass_	0.0015	0.0012	0.0024	0.0018	**0.006**	**0.024**	0.240
*θ*_mass_	0.014	0.011	0.026	0.019	**0.001**	**0.009**	0.133
*A*_max_ (μmol m^−2^ s^−1^)	28.1	27.6	24.2	24.5	**0.028**	0.958	0.728
*V*_*c*max_ (μmol m^−2^ s^−1^)	120.0	105.7	92.0	89.5	0.116	0.490	0.621
*J* (μmol m^−2^ s^−1^)	154.6	142.1	134.9	130.8	0.181	0.438	0.684
TPU (μmol m^−2^ s^−1^)	9.4	8.9	8.1	7.9	**0.053**	0.478	0.755
*A*_max_mass_ (μmol g^−1^ s^−1^)	0.57	0.46	0.84	0.62	**0.008**	**0.021**	0.273
*V*_*c*max_mass_ (μmol g^−1^ s^−1^)	2.5	1.8	3.2	2.3	0.103	**0.050**	0.725
*J*_mass_ (μmol g^−1^ s^−1^)	3.2	2.4	4.6	3.3	**0.013**	**0.020**	0.365
TPU_mass_ (μmol g^−1^ s^−1^)	0.19	0.15	0.28	0.20	**0.010**	**0.016**	0.281

*Note:* The subscript “mass” denotes expression per unit leaf mass instead of leaf area. Cucumber plants were grown for 4 weeks after transplanting under four treatments: SN × aCO_2_ (sinusoidal light + ambient CO_2_), SN × eCO_2_ (sinusoidal light + elevated CO_2_), FL × aCO_2_ (fluctuating light + ambient CO_2_), and FL × eCO_2_ (fluctuating light + elevated CO_2_). Data represent means of two experiments with 2–3 replicate plants per experiment. *p* values of light pattern (*L*), CO_2_, and their interaction (*L* × CO_2_) are shown, with significant effects (*p* < 0.1) in bold.

After the step increase from a low (120 μmol m^−2^ s^−1^) to a high light intensity (1200 μmol m^−2^ s^−1^), the photosynthetic induction state at 60 s (IS_60_) and the time to reach 50% and 90% of induction state (*T*_50_ and *T*_90_) were not significantly different between the treatments (Table S2). At a[CO_2_], *T*_50_ and *T*_90_ tended to be higher under FL than SN while at e[CO_2_], both tended to decrease, more strongly so under FL. FL‐grown plants showed significantly faster stomatal movement (i.e., lower *τ*_induction_ and *τ*_reduction_) compared to SN‐grown plants at both [CO_2_] (Table S2). The diurnal quantum yield of CO_2_ assimilation (*ɸ*_CO2_), measured at the respective growth conditions, tended to be higher under FL at e[CO_2_] compared to other treatments (Figure 3G). The operating respiration rate under the respective growth conditions (*R*_op_) was significantly lower under FL than SN at both CO_2_ concentrations (Figure 3H). The integrated *A*_diurnal_ over the 14 h measurement period was substantially reduced under FL compared to SN by 32% under both CO_2_ treatments (Figure 3I). Also, *A*_diurnal_ was significantly increased under e[CO_2_] compared to a[CO_2_] by 12% in both light treatments (Figure 3I).

### 3.3 Leaf Carbohydrate, Carbon, and Nitrogen Contents

**FIGURE 4:**
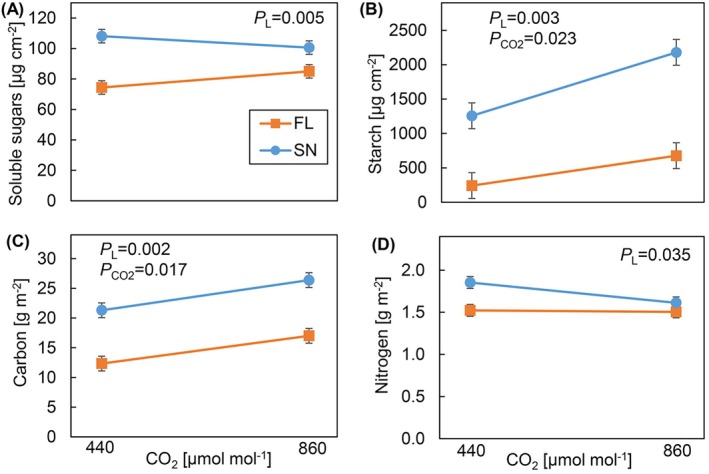
Leaf carbohydrate, carbon, and nitrogen contents per unit leaf area of cucumber plants grown under different light patterns and [CO_2_]. (A) Soluble sugars (sum of glucose, fructose sucrose, and stachyose), (B) starch, (C) carbon, (D) nitrogen. Cucumber plants were grown under four treatments: SN × aCO_2_ (sinusoidal light + ambient CO_2_), SN × eCO_2_ (sinusoidal light + elevated CO_2_), FL × aCO_2_ (fluctuating light + ambient CO_2_), and FL × eCO_2_ (fluctuating light + elevated CO_2_). Data represent means ± SEM based on two experiments each with 2–3 replicate plants per experiment. *p* value of the main effect of light pattern (*P*_*L*_) and CO_2_ (*P*_CO2_) is shown, when *p* < 0.1.

FL‐grown plants showed strongly reduced carbohydrate, carbon, and nitrogen contents per unit leaf area compared to SN‐grown plants, at both CO_2_ concentrations (Figure 4). The starch content was 73% lower in FL‐grown plants than SN‐grown plants in both CO_2_ concentrations (Figure 4B). e[CO_2_] significantly increased starch content (by 90%) compared with a[CO_2_] in both light treatments. FL‐grown plants had significantly lower carbon and nitrogen compared to SN‐grown plants in both CO_2_ concentrations (Figure 4C,D). e[CO_2_] significantly increased carbon content compared with a[CO_2_] (Figure 4C).

Per unit dry weight, the soluble sugar concentration was significantly higher under FL at both CO_2_ concentrations, compared to SN‐grown plants (Figure S8A). Compared with a[CO_2_], e[CO_2_] reduced the soluble sugars per unit dry weight (Figure S8A). However, the starch content per unit dry weight was significantly lower under FL than SN‐grown plants in both CO_2_ concentrations (Figure S8B). e[CO_2_] significantly increased starch contents under both light patterns compared with a[CO_2_] (Figure S8B). FL‐grown plants had significantly lower carbon content per unit dry weight, but higher nitrogen content compared to SN‐grown plants (Figure S8C,D). e[CO_2_] significantly reduced nitrogen content per unit dry weight compared with a[CO_2_] (Figure S8D).

### 3.4 Leaf Pigmentation and Leaf Light Absorptance

**FIGURE 5:**
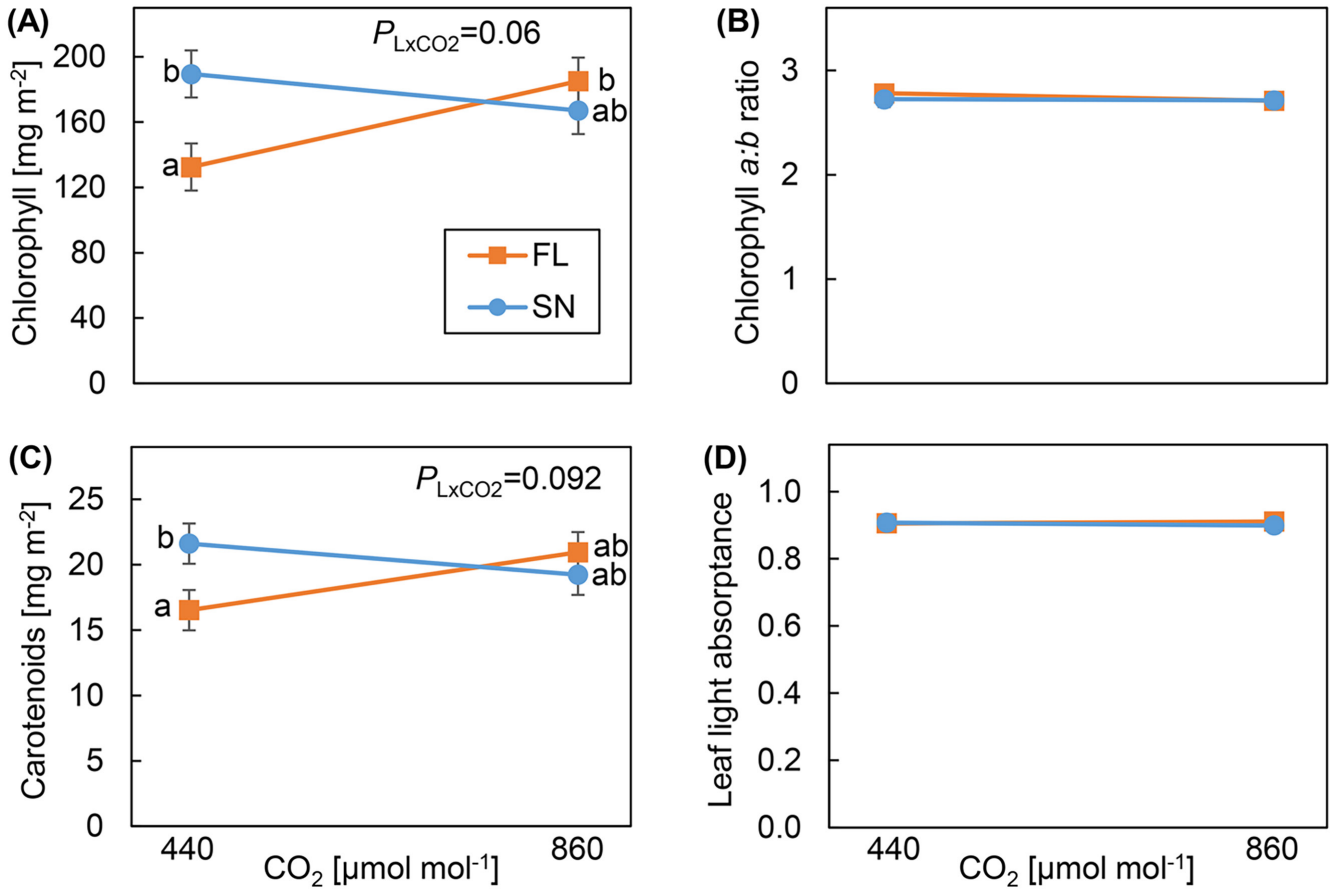
Leaf pigmentation per unit leaf area and leaf light absorptance of cucumber plants grown under different light patterns and [CO_2_]. (A) Chlorophyll content, (B) chlorophyll *a*:*b* ratio, (C) carotenoid content, (D) leaf light absorptance. Cucumber plants were grown under four treatments: SN × aCO_2_ (sinusoidal light + ambient CO_2_), SN × eCO_2_ (sinusoidal light + elevated CO_2_), FL × aCO_2_ (fluctuating light + ambient CO_2_), and FL × eCO_2_ (fluctuating light + elevated CO_2_). Data represent means ± SEM based on two experiments each with 2–3 replicate plants per experiment. *p* value of the interaction effect of light pattern and CO_2_ (*P*_*L*×CO2_) is shown, when *p* < 0.1. Different letters indicate significant differences between the treatments.

Light and CO_2_ treatments had interactive effects on leaf pigmentation: Although at a[CO_2_], FL‐grown plants had significantly lower chlorophyll and carotenoid contents (per unit leaf area) than SN‐grown plants, at e[CO_2_] these were similar between the light patterns (Figure 5A,C). Although concentrations of these pigments increased at e[CO_2_] under FL, they tended to decrease under SN. A similar trend was seen in chlorophyll *a* and *b* (Figure S9A,B). Per unit dry weight, leaf pigment contents were significantly higher under FL compared to SN at both [CO_2_] concentrations (Figure S9C–F). There were no treatment effects in chlorophyll *a*:*b* ratio and leaf light absorptance (Figure 5B,D). Although leaf light reflectance was significantly lower under FL‐grown plants compared to SN‐grown plants at both CO_2_ concentrations, no treatment effects were observed for leaf light transmittance (Figure S9G,H).

### 3.5 Stomatal Traits

**FIGURE 6:**
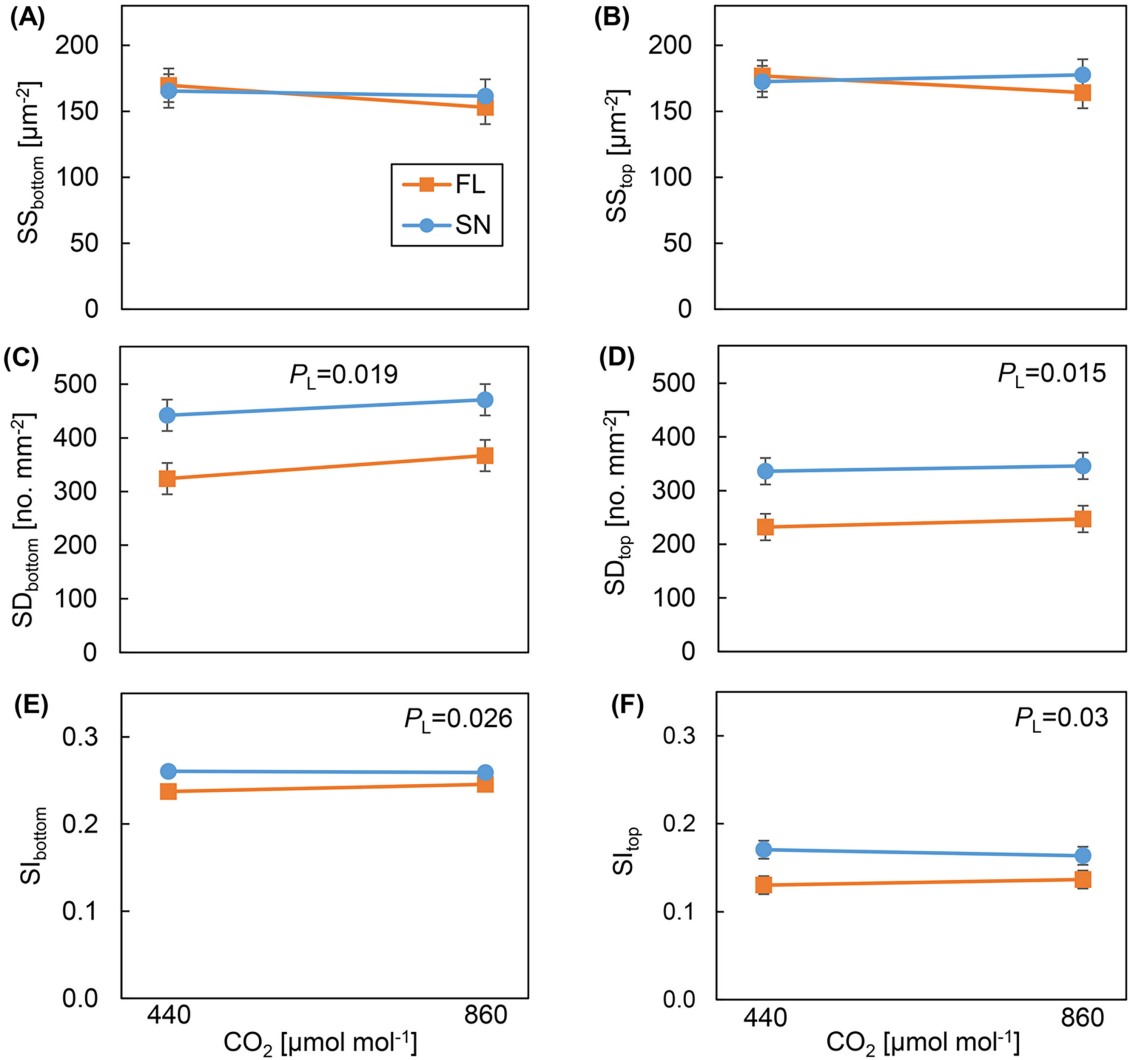
Traits describing stomatal morphology of cucumber leaves grown under different light patterns and [CO_2_]. (A, B) Abaxial and adaxial stomatal size (SS_bottom_, SS_top_), (C, D) abaxial and adaxial stomatal density (SD_bottom_, SD_top_), (E, F) abaxial and adaxial stomatal index (SI_bottom_, SI_top_). Cucumber plants were grown under four treatments: SN × aCO_2_ (sinusoidal light + ambient CO_2_), SN × eCO_2_ (sinusoidal light + elevated CO_2_), FL × aCO_2_ (fluctuating light + ambient CO_2_), and FL × eCO_2_ (fluctuating light + elevated CO_2_). Data represent means ± SEM based on two experiments each with three replicate plants per experiment. *p* value of the main effect of light pattern (*P*_*L*_) is shown, when *p* < 0.1.

FL‐grown plants had significantly lower stomatal density on both leaf surfaces and at both [CO_2_], than SN‐grown plants (Figure 6C,D). Similar results were observed for stomatal index (Figure 6E,F). There were no treatment effects on stomatal size (Figure 6A,B), and growth [CO_2_] did not affect any stomatal trait.

## 4 Discussion

Even though plants often grow under FL as well as more slowly changing SN light, and will likely grow under e[CO_2_] in the future due to climate change, very little is known about their combined effects on plant physiology and growth. Understanding how plants acclimate to FL and e[CO_2_] would allow us to predict future crop productivity under rising [CO_2_] in the field and the impact of reducing [CO_2_] in greenhouse crop production to meet sustainability targets. Therefore, we analyzed the acclimatory mechanisms of cucumber to a combination of FL and e[CO_2_], as compared to both SN and a[CO_2_]. Plants acclimated to FL largely by increasing leaf area and reducing leaf density, causing a 70% increase in SLA under FL compared to SN (Figure 1F,H). Under FL at a[CO_2_], morphological changes such as increases in stem length and leaf area (both of which may increase the area of photosynthesizing tissue) did not fully mitigate the reduced diurnal photosynthetic efficiency that likely led to the 25% reduction in dry mass compared to SN (Figures 1B,D,E and 3I). e[CO_2_] increased shoot biomass, photosynthetic efficiency, and tended to enhance leaf area, leading to a larger growth stimulation in FL than SN plants (Figures 1B,E and 3I), but this did not fully prevent a growth reduction under FL.

### 4.1 Changes in SLA Are Driven by Changes in Leaf Density Rather Than Thickness

SLA is highly responsive to light intensity and influences photosynthesis and growth (Poorter et al. 2013). In fast‐growing species like cucumber, SLA is highly plastic, with strong responses to the environment: examples include light intensity (~79% change; Trouwborst et al. 2011), light spectrum (~27%–56%; Kang et al. 2021), temperature (131%; Savvides et al. 2017), and [CO_2_] (~25%; Agüera et al. 2006). The 70% increase in SLA upon FL acclimation at a[CO_2_] (Figure 1F) was much larger than that observed in previous studies with FL (no changes; Kubásek et al. 2013, 7%–27% across studies: Morales and Kaiser 2020; Vialet‐Chabrand et al. 2017; Zhang et al. 2020), suggesting that the response of SLA to FL is species and/or environment specific. There is considerable debate as to whether leaf thickness or density explains the variation in SLA (Witkowski and Lamont 1991; Poorter et al. 2009; Xiong et al. 2015; Puglielli et al. 2017; Ye et al. 2020). In our case, the difference in SLA between FL and SN—70% at a[CO_2_] and 50% at e[CO_2_]—was mainly driven by leaf density rather than by leaf thickness, as there was no significant difference in leaf thickness between the treatments (Figures 1F–H and 2). The higher SLA under FL was mostly due to lower leaf density (Figure 1F,H). This observation in cucumber grown under FL differed from previous observations in Arabidopsis and tomato ( *Solanum lycopersicum* ), wherein changes in palisade and/or spongy mesophyll cell length caused an increase in SLA (Vialet‐Chabrand et al. 2017; Zhang et al. 2020). e[CO_2_] acclimation, on the other hand, often reduces SLA (Ainsworth et al. 2002; Leakey et al. 2002; Long et al. 2004) and is attributed to increases in leaf thickness (Zheng et al. 2019) or density (cell wall thickness and starch content; Mizokami et al. 2019), or both (Poorter et al. 2022). In our study, the reduction in SLA due to e[CO_2_] acclimation was attributed to higher leaf density rather than leaf thickness (Figure 1F–H).

Changes in leaf density could be attributed to leaf chemical composition such as TNCs, total structural carbohydrates (TSCs), nitrogen, and chlorophyll; or changes in cell size and number, the proportion of intercellular air spaces, cell wall thickness, and cell organelles such as mitochondria and chloroplasts (Poorter et al. 2009; Villar et al. 2013; Ren et al. 2019). While we observed lower soluble sugar and starch contents (TNCs) under FL (Figure 4A,B; also observed by Wei et al. 2021), TNCs were accumulated under e[CO_2_] (Figure 4A,B; also observed by Ainsworth and Long 2005; Poorter et al. 2022; Teng et al. 2006). Accounting for the contribution of TNCs to variation in SLA, SLA minus TNCs was still higher under FL at both [CO_2_] levels (Figure S6C). This suggests that while most variation in SLA was explained by TNC accumulation (Figure 2D)—and especially starch among the TNCs (Figure 2C)—other factors influenced SLA as well. Moreover, the presence of a lower number of organelles and proteins as indicated by lower *A* and *R*_op_ as well as lower chlorophyll and nitrogen per unit leaf area could also explain the lower density of FL leaves, while the opposite was true for SN leaves (Figures 3A,E,H,I, 4D, and 5A). Furthermore, the considerable difference in shoot FW and DW in FL‐grown plants (Figure 1A–C), indicating a higher water content in FL‐grown plants, could indicate the presence of larger vacuoles, which have lower densities compared to plastids (Poorter et al. 2009). Under FL, this strategy of making less dense leaves would mean a lower carbon investment per area, in turn allowing for larger and more numerous leaves for the same overall carbon cost. Indeed, larger leaves with lower density in FL‐grown plants (Figure 1E,H) likely enabled increased light capture during periods of low light intensity and with lower *R*_op_, which is indicative of lower maintenance costs. These adjustments under FL acclimation may be a fitness advantage under the FL pattern used here, where light intensity was often low (i.e., light intensity was at 120 μmol m^−2^ s^−1^ for 9.7 h out of the 16 h photoperiod in the FL treatments; Table S3).

### 4.2 Under a[CO_2_ ], FL Negatively Impacted Photosynthesis and Growth

In line with our hypothesis, FL negatively impacted cucumber dry mass, and the reduced photosynthetic efficiency under FL likely explains this reduction (Figures 1B and 3E,I), in line with previous findings (Kubásek et al. 2013; Vialet‐Chabrand et al. 2017; Morales and Kaiser 2020). The carbon gain of plants under FL is expected to be lower due to the nonlinear response of *A* to PPFD, the slow induction of *A* to PPFD increase (Figure 3A and Figure S7E; Morales and Kaiser 2020), and slow relaxation of NPQ after a PPFD decrease. We did not observe significant differences between FL and SN during the induction of photosynthesis (Table S2). However, we observed a diurnal decline in photosynthetic efficiency that was stronger under FL compared to SN (Figure 3G), confirming previous results (Vialet‐Chabrand et al. 2017). Several previous studies found that acclimation to FL did not result in faster kinetics of *A* upon step changes in PPFD (Sims and Pearcy 1993; Leakey et al. 2003; Kaiser et al. 2018). However, in Arabidopsis, growth under FL impacted the rapidity of *A* and *g*_*s*_ responses, and the observed differences were dependent on growth light patterns and time of day (Matthews et al. 2018). In this study, we did not observe different rapidity of response for *A*, but stomatal opening and closing were faster in FL than SN grown leaves (Table S2). This faster response of *g*_*s*_ only had a minor effect on the kinetics of *A* induction, likely because initial *g*_*s*_ was already high (Figure S7E,F and Table S2). Kaiser et al. (2016) observed that an initial *g*_*s*_ above a threshold value did not limit photosynthetic induction. It is intriguing that, despite having lower stomatal densities, FL‐grown plants showed faster stomatal kinetics (Figure 6C,D and Table S2). This lower stomatal density was likely the result of stronger leaf expansion that also reduced vein density, but only by 6% compared to SN‐grown plants (Figures 1I and 6C,D). This suggested that the spatial organization of veins and stomata was more optimal under FL to provide water for changes in transpiration and guard cell turgor (Lawson et al. 1998; Lawson and Weyers 1999; Fiorin et al. 2016).

In this study, the lower photosynthetic efficiency observed during the day could have been compensated by a larger leaf area. In cucumber, *A*_diurnal_ was more strongly reduced (by 32%) under FL compared to SN, resulting in a 73% reduction in starch, but only a 25% reduction in dry mass (Figures 1B, 3I, and 4B). This suggests that the 28% increase in leaf area under FL increased light capture and total carbon gain of the plant, partially compensating for lower photosynthetic efficiency per unit leaf area (Figures 1E and 3I). Furthermore, making less dense but larger leaves not only ensured lower construction and maintenance costs but also has implications for leaf chemical composition (as discussed earlier) and on photosynthesis. The higher photosynthetic capacities and nitrogen content per unit mass (Figure 3B,F and Figure S8D; Table 1) indicated that FL‐grown plants might invest less in leaf structure and maintain *A* per unit area by increasing the number of photosynthetic organelles and proteins per cell. Additionally, mitochondrial respiration measured under growth conditions was lower in FL‐grown leaves (Figure 3H), which may partially offset lower photosynthesis efficiency. Altogether, morphological and anatomical adjustments partially compensated for the reductions in leaf photosynthesis under fluctuating compared to SN light.

### 4.3 e[CO_2_ ] Partially Mitigated the Negative Effect of FL on Cucumber Growth

e[CO_2_] had larger effects on plant growth under FL (e.g., increased shoot biomass). Long et al. (2004) observed that growth under e[CO_2_] increased both leaf area and *A*, likely resulting in a positive feedback on whole‐plant *A* and growth. Moreover, Leakey et al. (2002) showed stronger enhancement effects of e[CO_2_] under FL than under constant light. We observed a similar increase in photosynthesis during the day, increased shoot biomass, and a tendency of increased leaf area under e[CO_2_], which were stronger under FL (Figures 1B,E and 3I). The additional TNCs produced due to increased *A*_diurnal_ under e[CO_2_] at FL (Figures 3I and 4A,B) were invested in shoots, which further enhanced light capture, as discussed previously. Interestingly, acclimation to e[CO_2_] neither affected steady‐state nor dynamic photosynthesis traits per leaf area (Table 1 and Table S2), confirming results for cucumber by Zhang et al. (2024). However, steady‐state photosynthetic traits per unit mass were lower under e[CO_2_] acclimation (Figure 3B,F and Table 1), and this was mostly due to accumulated TNCs contributing to denser leaves and thereby lower SLA (Figures 1F,H, 2D, and 4B). There was no significant difference between e[CO_2_] and a[CO_2_] in other traits such as stomatal density, vein density, and leaf nitrogen content (Figures 1I, 4D, and 6C,D). Overall, e[CO_2_] mitigated several negative effects of FL on plant growth.

## 5 Conclusions

We showed here that cucumber acclimated to FL intensities, as compared to a slowly changing SN light intensity, through large adjustments of leaf anatomy as well as relatively smaller adjustments to stem height, leaf pigmentation, and stomatal morphology. The adjustments seen to SLA were much larger than previously reported for other plant species, likely because of the large environmental plasticity of cucumber. Under FL, larger leaves with lower density had lower maintenance costs and partially compensated for reductions in leaf photosynthesis efficiency and growth compared to SN. Growth under e[CO_2_] partially offset some of the negative impacts of FL, especially with regard to SLA and photosynthetic efficiency. In contrast to morphology, steady‐state and dynamic photosynthesis traits were relatively unaffected by acclimation to either FL or [CO_2_]. Our results indicate that fast‐growing species such as cucumber acclimate to FL and different [CO_2_] by changes to leaf anatomy and plant morphology rather than photosynthetic capacity.

## Author Contributions

S.S., S.V.‐C., E.K., and L.F.M.M. designed the study. S.S. collected and analyzed data. S.R.B. helped with gas exchange protocols and data collection. J.O. helped with microscopy work. S.S. wrote the manuscript with the help of E.K. and S.V.‐C. and was revised by the rest of the authors.

## Supporting information


**Table S1:** Growth traits of cucumber plants grown under the four treatments.
**Table S2:** Dynamic photosynthesis parameters of cucumber leaves grown under the four treatments.
**Table S3:** Key characteristics of the light patterns used in this study.
**Figure S1:** Leaf support structure used to make leaf #6 horizontal during growth.
**Figure S2:** Light treatments used for cucumber plant growth.
**Figure S3:** Duration per category of light intensity during sinusoidal and fluctuating light patterns.
**Figure S4:** Images of leaf veins of cucumber leaves grown under the four treatments.
**Figure S5:** Leaf cross‐section images of cucumber leaves grown under the four treatments.
**Figure S6:** Leaf anatomical properties of cucumber leaves grown under the four treatments.
**Figure S7:** Steady‐state and dynamic photosynthesis of cucumber leaves grown under the four treatments.
**Figure S8:** Leaf carbohydrate, carbon, and nitrogen content per unit leaf dry weight of cucumber leaves grown under the four treatments.
**Figure S9:** Leaf pigment content and optical properties of cucumber leaves grown under the four treatments.

## Data Availability

The data that support the findings of this study are available from the corresponding authors upon reasonable request.

## References

[ppl70436-bib-0001] Agüera, E. , D. Ruano , P. Cabello , and P. de la Haba . 2006. “Impact of Atmospheric CO_2_ on Growth, Photosynthesis and Nitrogen Metabolism in Cucumber ( *Cucumis sativus* L.) Plants.” Journal of Plant Physiology 163, no. 8: 809–817. 10.1016/j.jplph.2005.08.010. 16777528

[ppl70436-bib-0002] Ainsworth, E. A. , P. A. Davey , C. J. Bernacchi , et al. 2002. “A Meta‐Analysis of Elevated [CO_2_] Effects on Soybean ( *Glycine max* ) Physiology, Growth and Yield.” Global Change Biology 8, no. 8: 695–709.

[ppl70436-bib-0003] Ainsworth, E. A. , and S. P. Long . 2005. “What Have We Learned From 15 Years of Free‐Air CO_2_ Enrichment (FACE)? A Meta‐Analytic Review of the Responses of Photosynthesis, Canopy Properties and Plant Production to Rising CO_2_ .” New Phytologist 165, no. 2: 351–372. 15720649 10.1111/j.1469-8137.2004.01224.x

[ppl70436-bib-0004] Ainsworth, E. A. , and A. Rogers . 2007. “The Response of Photosynthesis and Stomatal Conductance to Rising [CO_2_]: Mechanisms and Environmental Interactions.” Plant, Cell & Environment 30, no. 3: 258–270. 10.1111/j.1365-3040.2007.01641.x. 17263773

[ppl70436-bib-0005] Allen, L. H. , B. A. Kimball , J. A. Bunce , et al. 2020. “Fluctuations of CO_2_ in Free‐Air CO_2_ Enrichment (FACE) Depress Plant Photosynthesis, Growth, and Yield.” Agricultural and Forest Meteorology 284: 107899. 10.1016/j.agrformet.2020.107899.

[ppl70436-bib-0006] Alter, P. , A. Dreissen , F. L. Luo , and M. Shizue . 2012. “Acclimatory Responses of Arabidopsis to Fluctuating Light Environment: Comparison of Different Sunfleck Regimes and Accessions.” Photosynthesis Research 113: 221–237. 10.1007/s11120-012-9757-2. 22729524 PMC3430843

[ppl70436-bib-0007] Athanasiou, K. , B. C. Dyson , R. E. Webster , and G. N. Johnson . 2010. “Dynamic Acclimation of Photosynthesis Increases Plant Fitness in Changing Environments.” Plant Physiology 152, no. 1: 366–373. 10.1104/pp.109.149351. 19939944 PMC2799370

[ppl70436-bib-0008] Becklin, K. M. , J. K. Ward , and D. A. Way , eds. 2021. Photosynthesis, Respiration, and Climate Change. Springer.

[ppl70436-bib-0009] Bhuiyan, R. , and M. W. Van Iersel . 2021. “Only Extreme Fluctuations in Light Levels Reduce Lettuce Growth Under Sole Source Lighting.” Frontiers in Plant Science 12: 619973. 10.3389/fpls.2021.619973. 33584773 PMC7875872

[ppl70436-bib-0010] Blom, T. J. , and Y. Zheng . 2009. “The Response of Plant Growth and Leaf Gas Exchange to the Speed of Lamp Movement in a Greenhouse.” Scientia Horticulturae 119, no. 2: 188–192. 10.1016/j.scienta.2008.07.014.

[ppl70436-bib-0011] Brodribb, T. J. , and G. J. Jordan . 2011. “Water Supply and Demand Remain Balanced During Leaf Acclimation of *Nothofagus cunninghamii* Trees.” New Phytologist 192, no. 2: 437–448. 10.1111/j.1469-8137.2011.03795.x. 21679190

[ppl70436-bib-0012] Burgess, A. J. , M. Durand , J. A. Gibbs , R. Retkute , T. M. Robson , and E. H. Murchie . 2021. “The Effect of Canopy Architecture on the Patterning of ‘Windflecks’ Within a Wheat Canopy.” Plant, Cell & Environment 44, no. 11: 3524–3537. 10.1111/pce.14168. 34418115

[ppl70436-bib-0013] Burgess, A. J. , R. Retkute , M. P. Pound , et al. 2015. “High‐Resolution Three‐Dimensional Structural Data Quantify the Impact of Photoinhibition on Long‐Term Carbon Gain in Wheat Canopies in the Field.” Plant Physiology 169, no. 2: 1192–1204. 10.1104/pp.15.00722. 26282240 PMC4587458

[ppl70436-bib-0014] Carins Murphy, M. R. , G. J. Jordan , and T. J. Brodribb . 2012. “Differential Leaf Expansion Can Enable Hydraulic Acclimation to Sun and Shade.” Plant, Cell & Environment 35, no. 8: 1407–1418. 10.1111/j.1365-3040.2012.02498.x. 22339445

[ppl70436-bib-0015] Chen, G. Y. , Z. H. Yong , Y. Liao , et al. 2005. “Photosynthetic Acclimation in Rice Leaves to Free‐Air CO_2_ Enrichment Related to Both Ribulose‐1,5‐Bisphosphate Carboxylation Limitation and Ribulose‐1,5‐Bisphosphate Regeneration Limitation.” Plant and Cell Physiology 46, no. 7: 1036–1045. 10.1093/pcp/pci113. 15840641

[ppl70436-bib-0016] Chen, T.‐W. , H. Stützel , and K. Kahlen . 2018. “High Light Aggravates Functional Limitations of Cucumber Canopy Photosynthesis Under Salinity.” Annals of Botany 121, no. 5: 797–807. 10.1093/aob/mcx100. 29028871 PMC5906908

[ppl70436-bib-0017] Curtis, P. S. , and X. Wang . 1998. “A Meta‐Analysis of Elevated CO_2_ Effects on Woody Plant Mass, Form, and Physiology.” Oecologia 113: 299–313. 28307814 10.1007/s004420050381

[ppl70436-bib-0018] Drag, D. W. , R. Slattery , M. Siebers , E. H. Delucia , D. R. Ort , and C. J. Bernacchi . 2020. “Soybean Photosynthetic and Biomass Responses to Carbon Dioxide Concentrations Ranging From Pre‐Industrial to the Distant Future.” Journal of Experimental Botany 71, no. 12: 3690–3700. 10.1093/jxb/eraa133. 32170296 PMC7475242

[ppl70436-bib-0019] Durand, M. , B. Matule , A. J. Burgess , and T. M. Robson . 2021. “Sunfleck Properties From Time Series of Fluctuating Light.” Agricultural and Forest Meteorology 308: 108554. 10.1016/j.agrformet.2021.108554.

[ppl70436-bib-0020] Fiorin, L. , T. J. Brodribb , and T. Anfodillo . 2016. “Transport Efficiency Through Uniformity: Organization of Veins and Stomata in Angiosperm Leaves.” New Phytologist 209, no. 1: 216–227. 10.1111/nph.13577. 26224215

[ppl70436-bib-0021] Gjindali, A. , H. A. Herrmann , J. M. Schwartz , G. N. Johnson , and P. I. Calzadilla . 2021. “A Holistic Approach to Study Photosynthetic Acclimation Responses of Plants to Fluctuating Light.” Frontiers in Plant Science 12: 668512. 10.3389/fpls.2021.668512. 33936157 PMC8079764

[ppl70436-bib-0022] Hogewoning, S. W. , P. Douwstra , G. Trouwborst , W. Van Ieperen , and J. Harbinson . 2010. “An Artificial Solar Spectrum Substantially Alters Plant Development Compared With Usual Climate Room Irradiance Spectra.” Journal of Experimental Botany 61, no. 5: 1267–1276. 10.1093/jxb/erq005. 20202994

[ppl70436-bib-0023] Intergovernmental Panel on Climate (IPCC) . 2023. “Technical Summary.” In Climate Change 2021—The Physical Science Basis: Working Group I Contribution to the Sixth Assessment Report of the Intergovernmental Panel on Climate Change, 35–144. Cambridge University Press. 10.1017/9781009157896.002.

[ppl70436-bib-0024] Kaiser, E. , S. Matsubara , J. Harbinson , E. Heuvelink , and L. F. M. Marcelis . 2018. “Acclimation of Photosynthesis to Lightflecks in Tomato Leaves: Interaction With Progressive Shading in a Growing Canopy.” Physiologia Plantarum 162, no. 4: 506–517. 10.1111/ppl.12668. 29125181

[ppl70436-bib-0025] Kaiser, E. , A. Morales , J. Harbinson , E. Heuvelink , A. E. Prinzenberg , and L. F. M. Marcelis . 2016. “Metabolic and Diffusional Limitations of Photosynthesis in Fluctuating Irradiance in *Arabidopsis thaliana* .” Scientific Reports 6, no. 1: 31252. 10.1038/srep31252. 27502328 PMC4977489

[ppl70436-bib-0026] Kaiser, E. , T. Ouzounis , H. Giday , R. Schipper , E. Heuvelink , and L. F. M. Marcelis . 2019. “Adding Blue to Red Supplemental Light Increases Biomass and Yield of Greenhouse‐Grown Tomatoes, but Only to an Optimum.” Frontiers in Plant Science 9: 417174. 10.3389/fpls.2018.02002. PMC6339924 30693012

[ppl70436-bib-0027] Kang, C. , Y. Zhang , R. Cheng , E. Kaiser , Q. Yang , and T. Li . 2021. “Acclimating Cucumber Plants to Blue Supplemental Light Promotes Growth in Full Sunlight.” Frontiers in Plant Science 12: 782465. 10.3389/fpls.2021.782465. 34912362 PMC8668241

[ppl70436-bib-0028] Kono, M. , and I. Terashima . 2014. “Long‐Term and Short‐Term Responses of the Photosynthetic Electron Transport to Fluctuating Light.” Journal of Photochemistry and Photobiology, B: Biology 137: 89–99. 10.1016/j.jphotobiol.2014.02.016. 24776379

[ppl70436-bib-0029] Kubásek, J. , O. Urban , and J. Šantrůček . 2013. “C4 Plants Use Fluctuating Light Less Efficiently Than Do C3 Plants: A Study of Growth, Photosynthesis and Carbon.” Physiologia Plantarum 149, no. 4: 528–539. 10.1111/ppl.12057. 23550566

[ppl70436-bib-0030] Larsen, D. H. , H. Li , A. C. DeVan Peppel , C. C. S. Nicole , L. F. M. Marcelis , and E. J. Woltering . 2022. “High Light Intensity at End‐Of‐Production Improves the Nutritional Value of Basil but Does Not Affect Postharvest Chilling Tolerance.” Food Chemistry 369: 130913. 10.1016/j.foodchem.2021.130913. 34481404

[ppl70436-bib-0031] Lawson, T. , and J. Weyers . 1999. “Spatial and Temporal Variation in Gas Exchange Over the Lower Surface of *Phaseolus vulgaris* L. Primary Leaves.” Journal of Experimental Botany 50, no. 337: 1381–1391. 10.1093/jxb/50.337.1381.

[ppl70436-bib-0032] Lawson, T. , J. Weyers , and R. A. Brook . 1998. “The Nature of Heterogeneity in the Stomatal Behaviour of *Phaseolus vulgaris* L. Primary Leaves.” Journal of Experimental Botany 49, no. 325: 1387–1395.

[ppl70436-bib-0033] Leakey, A. D. B. , M. C. Press , and J. D. Scholes . 2003. “Patterns of Dynamic Irradiance Affect the Photosynthetic Capacity and Growth of Dipterocarp Tree Seedlings.” Oecologia 135: 184–193. 10.1007/s00442-003-1178-7. 12698339

[ppl70436-bib-0034] Leakey, A. D. B. , M. C. Press , J. D. Scholes , and J. R. Watling . 2002. “Relative Enhancement of Photosynthesis and Growth at Elevated CO_2_ Is Greater Under Sunflecks Than Uniform Irradiance in a Tropical Rain Forest Tree Seedling.” Plant, Cell & Environment 25, no. 12: 1701–1714.

[ppl70436-bib-0035] Lichtenthaler, H. K. 1987. “Chlorophylls Carotenoids: Pigments of Photosynthetic Biomembranes.” In Methods in Enzymology, 350–382. Academic Press.

[ppl70436-bib-0036] Liu, Y. , W. Dawson , D. Prati , E. Haeuser , Y. Feng , and M. van Kleunen . 2016. “Does Greater Specific Leaf Area Plasticity Help Plants to Maintain a High Performance When Shaded?” Annals of Botany 118, no. 7: 1329–1336. 10.1093/aob/mcw180. 27594648 PMC5155598

[ppl70436-bib-0037] Lobo, F. d. A. , M. P. de Barros , H. J. Dalmagro , et al. 2013. “Fitting Net Photosynthetic Light‐Response Curves With Microsoft Excel—A Critical Look at the Models.” Photosynthetica 51, no. 3: 445–456. 10.1007/s11099-013-0045-y.

[ppl70436-bib-0038] Long, S. P. , E. A. Ainsworth , A. Rogers , and D. R. Ort . 2004. “Rising Atmospheric Carbon Dioxide: Plants FACE the Future.” Annual Review of Plant Biology 55: 591–628. 10.1146/annurev.arplant.55.031903.141610. 15377233

[ppl70436-bib-0039] Matthews, J. S. A. , S. Vialet‐Chabrand , and T. Lawson . 2018. “Acclimation to Fluctuating Light Impacts the Rapidity of Response and Diurnal Rhythm of Stomatal Conductance.” Plant Physiology 176, no. 3: 1939–1951. 10.1104/pp.17.01809. 29371250 PMC5841698

[ppl70436-bib-0040] Mizokami, Y. , D. Sugiura , C. K. A. Watanabe , E. Betsuyaku , and N. Inada . 2019. “Elevated CO_2_‐Induced Changes in Mesophyll Conductance and Anatomical Traits in Wild Type and Carbohydrate‐Metabolism Mutants of Arabidopsis.” Journal of Experimental Botany 70, no. 18: 4807–4818. 10.1093/jxb/erz208. 31056658 PMC6760322

[ppl70436-bib-0041] Morales, A. , and E. Kaiser . 2020. “Photosynthetic Acclimation to Fluctuating Irradiance in Plants.” Frontiers in Plant Science 11: 508216. 10.3389/fpls.2020.00268. PMC7105707 32265952

[ppl70436-bib-0042] Moualeu‐Ngangue, D. P. , T. Chen , and H. Stutzel . 2017. “A New Method to Estimate Photosynthetic Parameters Through Net Assimilation Rate À Intercellular Space CO_2_ Concentration (A − *C* _ *i* _) Curve and Chlorophyll Fluorescence Measurements.” New Phytologist 213: 1543–1554. 10.1111/nph.14260. 27768807

[ppl70436-bib-0043] Pao, Y.‐C. , T.‐W. Chen , D. P. Moualeu‐ngangue , and H. Stützel . 2019. “Environmental Triggers for Photosynthetic Protein Turnover Determine the Optimal Nitrogen Distribution and Partitioning in the Canopy.” Journal of Experimental Botany 70, no. 9: 2419–2433. 10.1093/jxb/ery308. 30124935 PMC6519421

[ppl70436-bib-0044] Pearcy, R. W. 1990. “Sunflecks and Photosynthesis in Plant Canopies.” Annual Review of Plant Biology 41, no. 1: 421–453.

[ppl70436-bib-0045] Pearcy, R. W. , J. P. Krall , and G. F. Sassenrath‐cole . 1996. “Photosynthesis in Fluctuating Light Environments.” In Photosynthesis and the Environment, 321–346. Dordrecht: Springer.

[ppl70436-bib-0046] Poorter, H. , N. P. R. Anten , and L. F. M. Marcelis . 2013. “Physiological Mechanisms in Plant Growth Models: Do We Need a Supra‐Cellular Systems Biology Approach?” Plant, Cell & Environment 36, no. 9: 1673–1690. 10.1111/pce.12123. 23611725

[ppl70436-bib-0047] Poorter, H. , O. Knopf , I. J. Wright , et al. 2022. “A Meta‐Analysis of Responses of C3 Plants to Atmospheric CO_2_: Dose–Response Curves for 85 Traits Ranging From the Molecular to the Whole‐Plant Level.” New Phytologist 233, no. 4: 1560–1596. 10.1111/nph.17802. 34657301

[ppl70436-bib-0048] Poorter, H. , H. Poorter , Ü. Niinemets , L. Poorter , I. J. Wright , and R. Villar . 2009. “Causes and Consequences of Variation in Leaf Mass Per Area (LMA): A Meta‐Analysis.” New Phytologist 182, no. 3: 565–588. 10.1111/j.1469-8137.2009.02830.x. 19434804

[ppl70436-bib-0049] Porra, R. J. , W. A. Thompson , and P. E. Kriedemann . 1989. “Determination of Accurate Extinction Coefficients and Simultaneous Equations for Assaying Chlorophylls a and b Extracted With Four Different Solvents: Verification of the Concentration of Chlorophyll Standards by Atomic Absorption Spectroscopy.” Biochimica et Biophysica Acta (BBA)‐Bioenergetics 975, no. 3: 384–394.

[ppl70436-bib-0050] Puglielli, G. , L. Varone , L. Gratani , and R. Catoni . 2017. “Specific Leaf Area Variations Drive Acclimation of *Cistus salvifolius* in Different Light Environments.” Photosynthetica 55: 31–40. 10.1007/s11099-016-0235-5.

[ppl70436-bib-0051] Ren, T. , S. M. Weraduwage , and T. D. Sharkey . 2019. “Prospects for Enhancing Leaf Photosynthetic Capacity by Manipulating Mesophyll Cell Morphology.” Journal of Experimental Botany 70, no. 4: 1153–1165. 10.1093/jxb/ery448. 30590670

[ppl70436-bib-0052] Retkute, R. , S. E. Smith‐unna , R. W. Smith , et al. 2015. “Exploiting Heterogeneous Environments: Does Photosynthetic Acclimation Optimize Carbon Gain in Fluctuating Light?” Journal of Experimental Botany 66, no. 9: 2437–2447. 10.1093/jxb/erv055. 25788730 PMC4629418

[ppl70436-bib-0053] Ruiz‐vera, U. M. , A. P. De Souza , M. R. Ament , R. M. Gleadow , and D. R. Ort . 2021. “High Sink Strength Prevents Photosynthetic Down‐Regulation in Cassava Grown at Elevated CO_2_ Concentration.” Journal of Experimental Botany 72, no. 2: 542–560. 10.1093/jxb/eraa459. 33045084 PMC7853607

[ppl70436-bib-0054] Savvides, A. , D. Fanourakis , and W. Van Ieperen . 2012. “Co‐Ordination of Hydraulic and Stomatal Conductances Across Light Qualities in Cucumber Leaves.” Journal of Experimental Botany 63, no. 3: 1135–1143. 10.1093/jxb/err348. 22121201 PMC3276089

[ppl70436-bib-0055] Savvides, A. , W. van Ieperen , J. A. Dieleman , and L. F. M. Marcelis . 2017. “Phenotypic Plasticity to Altered Apical Bud Temperature in *Cucumis sativus* : More Leaves‐Smaller Leaves and Vice Versa.” Plant, Cell & Environment 40, no. 1: 69–79. 10.1111/pce.12835. 27640366

[ppl70436-bib-0056] Schöttler, M. A. , S. Z. Tóth , A. Boulouis , and S. Kahlau . 2015. “Photosynthetic Complex Stoichiometry Dynamics in Higher Plants: Biogenesis, Function, and Turnover of ATP Synthase and the Cytochrome *b* _6_ *f* Complex.” Journal of Experimental Botany 66, no. 9: 2373–2400. 10.1093/jxb/eru495. 25540437

[ppl70436-bib-0057] Schumann, T. , S. Paul , M. Melzer , P. Dörmann , and P. Jahns . 2017. “Plant Growth Under Natural Light Conditions Provides Highly Flexible Short‐Term Acclimation Properties Toward High Light Stress.” Frontiers in Plant Science 8: 681. 10.3389/fpls.2017.00681. 28515734 PMC5413563

[ppl70436-bib-0058] Sharkey, T. D. 2016. “What Gas Exchange Data Can Tell Us About Photosynthesis.” Plant, Cell & Environment 39, no. 6: 1161–1163. 10.1111/pce.12641. 26390237

[ppl70436-bib-0059] Sims, D. A. , and R. W. Pearcy . 1993. “Sunfleck Frequency and Duration Affects Growth Rate of the Understorey Plant, *Alocasia macrorrhiza* .” Functional Ecology 7: 683–689.

[ppl70436-bib-0060] Teng, N. , J. Wang , T. Chen , X. Wu , Y. Wang , and J. Lin . 2006. “Elevated CO_2_ Induces Physiological, Biochemical and Structural Changes in Leaves of *Arabidopsis thaliana* .” New Phytologist 172, no. 1: 92–103. 10.1111/j.1469-8137.2006.01818.x. 16945092

[ppl70436-bib-0061] Tomimatsu, H. , T. Sakata , H. Fukayama , and Y. Tang . 2019. “Short‐Term Effects of High CO_2_ Accelerate Photosynthetic Induction in *Populus koreana* × *trichocarpa* With Always‐Open Stomata Regardless of Phenotypic Changes in High CO_2_ Growth Conditions.” Tree Physiology 39, no. 3: 474–483. 10.1093/treephys/tpy078. 30053250

[ppl70436-bib-0062] Trouwborst, G. , S. W. Hogewoning , J. Harbinson , and W. Van Ieperen . 2011. “Photosynthetic Acclimation in Relation to Nitrogen Allocation in Cucumber Leaves in Response to Changes in Irradiance.” Physiologia Plantarum 142, no. 2: 157–169. 10.1111/j.1399-3054.2011.01456.x. 21320128

[ppl70436-bib-0063] Uhl, D. , and V. Mosbrugger . 1999. “Leaf Venation Density as a Climate and Environmental Proxy: A Critical Review and New Data.” Palaeogeography, Palaeoclimatology, Palaeoecology 149, no. 1–4: 15–26.

[ppl70436-bib-0064] van Westreenen, A. , N. Zhang , E. Kaiser , et al. 2023. “Rapid Irradiance Fluctuations Occur in a Greenhouse: Quantification and Implication.” Biosystems Engineering 235: 215–229. 10.1016/j.biosystemseng.2023.10.004.

[ppl70436-bib-0065] Vialet‐Chabrand, S. , J. S. A. Matthews , A. J. Simkin , C. A. Raines , and T. Lawson . 2017. “Importance of Fluctuations in Light on Plant.” Plant Physiology 173, no. 4: 2163–2179. 10.1104/pp.16.01767. 28184008 PMC5373038

[ppl70436-bib-0066] Villar, R. , J. Ruiz‐Robleto , J. L. Ubera , and H. Poorter . 2013. “Exploring Variation in Leaf Mass Per Area (LMA) From Leaf to Cell: An Anatomical Analysis of 26 Woody Species.” American Journal of Botany 100, no. 10: 1969–1980. 10.3732/ajb.1200562. 24107583

[ppl70436-bib-0067] Wall, S. , J. Cockram , S. Vialet‐Chabrand , J. Van Rie , A. Gallé , and T. Lawson . 2023. “The Impact of Growth at Elevated [CO_2_] on Stomatal Anatomy and Behavior Differs Between Wheat Species and Cultivars.” Journal of Experimental Botany 74, no. 9: 2860–2874. 10.1093/jxb/erad011. 36633860 PMC10134898

[ppl70436-bib-0068] Walters, R. G. 2005. “Towards an Understanding of Photosynthetic Acclimation.” Journal of Experimental Botany 56, no. 411: 435–447. 10.1093/jxb/eri060. 15642715

[ppl70436-bib-0069] Way, D. A. , and R. W. Pearcy . 2012. “Sunflecks in Trees and Forests: From Photosynthetic Physiology to Global Change Biology.” Tree Physiology 32, no. 9: 1066–1081. 10.1093/treephys/tps064. 22887371

[ppl70436-bib-0070] Wei, Z. , F. Duan , X. Sun , X. Song , and W. Zhou . 2021. “Leaf Photosynthetic and Anatomical Insights Into Mechanisms of Acclimation in Rice in Response to Long‐Term Fluctuating Light.” Plant, Cell & Environment 44, no. 3: 747–761. 10.1111/pce.13954. 33215722

[ppl70436-bib-0071] Witkowski, E. T. F. , and B. B. Lamont . 1991. “Leaf Specific Mass Confounds Leaf Density and Thickness.” Oecologia 88: 486–493. 28312617 10.1007/BF00317710

[ppl70436-bib-0072] Xiong, D. , T. Yu , T. Zhang , Y. Li , S. Peng , and J. Huang . 2015. “Leaf Hydraulic Conductance Is Coordinated With Leaf Morpho‐Anatomical Traits and Nitrogen Status in the Genus *Oryza* .” Journal of Experimental Botany 66, no. 3: 741–748. 10.1093/jxb/eru434. 25429002 PMC4321541

[ppl70436-bib-0073] Ye, M. , M. Wu , H. Zhang , Z. Zhang , and Z. Zhang . 2021. “High Leaf Vein Density Promotes Leaf Gas Exchange by Enhancing Leaf Hydraulic Conductance in *Oryza sativa* L. Plants.” Frontiers in Plant Science 12: 693815. 10.3389/fpls.2021.693815. 34759936 PMC8573028

[ppl70436-bib-0074] Ye, M. , Z. Zhang , G. Huang , Z. Xiong , S. Peng , and Y. Li . 2020. “High Leaf Mass Per Area *Oryza* Genotypes Invest More Leaf Mass to Cell Wall and Show a Low Mesophyll Conductance.” AoB Plants 12, no. 4: plaa028. 10.1093/AOBPLA/PLAA028. 32765824 PMC7396964

[ppl70436-bib-0075] Yin, Z. H. , and G. N. Johnson . 2000. “Photosynthetic Acclimation of Higher Plants to Growth in Fluctuating Light Environments.” Photosynthesis Research 63: 97–107. 16252168 10.1023/A:1006303611365

[ppl70436-bib-0076] Yu, L. , K. Fujiwara , and R. Matsuda . 2022. “Estimating Light Acclimation Parameters of Cucumber Leaves Using Time‐Weighted Averages of Daily Photosynthetic Photon Flux Density.” Frontiers in Plant Science 12: 809046. 10.3389/fpls.2021.809046. 35211135 PMC8860900

[ppl70436-bib-0077] Zhang, N. , S. R. Berman , T. van den Berg , Y. Chen , L. F. M. Marcelis , and E. Kaiser . 2024. “Biochemical Versus Stomatal Acclimation of Dynamic Photosynthetic Gas Exchange to Elevated CO_2_ in Three Horticultural Species With Contrasting Stomatal Morphology.” Plant, Cell & Environment 47: 4516–4529. 10.1111/pce.15043. 39011936

[ppl70436-bib-0078] Zhang, Y. , E. Kaiser , L. F. M. Marcelis , Q. Yang , and T. Li . 2020. “Salt Stress and Fluctuating Light Have Separate Effects on Photosynthetic Acclimation, but Interactively Affect Biomass.” Plant, Cell & Environment 43, no. 9: 2192–2206. 10.1111/pce.13810. 32463133

[ppl70436-bib-0079] Zheng, Y. , F. Li , L. Hao , et al. 2019. “Elevated CO_2_ Concentration Induces Photosynthetic Down‐Regulation With Changes in Leaf Structure, Non‐Structural Carbohydrates and Nitrogen Content of Soybean.” BMC Plant Biology 19: 1–18. 10.1186/s12870-019-1788-9. 31195963 PMC6567668

