# Supplementary material for: Elevated [CO2 ] Affected Fluctuating Light Acclimation in Cucumber Plants by Changes in Specific Leaf Area and Photosynthetic Efficiency
Source: Physiol Plant. 2025 Aug 5;177(4):e70436. doi: 10.1111/ppl.70436 (PMC12322873; doi:10.1111/ppl.70436)
Supplement: Supplementary file 1 — Table S1: Growth traits of cucumber plants grown under the four treatments. Table S2: Dynamic photosynthesis parameters of cucumber leaves grown under the four treatments. Table S3: Key characteristics of the light patterns used in this study. Figure S1: Leaf support structure used to make leaf #6 horizontal during growth. Figure S2: Light treatments used for cucumber plant growth. Figure S3: Duration per category of light intensity during sinusoidal and fluctuating light patterns. Figure S4: Images of leaf veins of cucumber leaves grown under the four treatments. Figure S5: Leaf cross‐section images of cucumber leaves grown under the four treatments. Figure S6: Leaf anatomical properties of cucumber leaves grown under the four treatments. Figure S7: Steady‐state and dynamic photosynthesis of cucumber leaves grown under the four treatments. Figure S8: Leaf carbohydrate, carbon, and nitrogen content per unit leaf dry weight of cucumber leaves grown under the four treatments. Figure S9: Leaf pigment content and optical properties of cucumber leaves grown under the four treatments. [file PPL-177-e70436-s001.pdf]

Elevated [CO_2] affected fluctuating light acclimation in cucumber by changes in specific leaf area and photosynthetic efficiency

Samikshya Shrestha[1], Sarah R. Berman[1], Joke Oosterkamp[1], Leo F.M. Marcelis[1], Elias Kaiser[1,2,*], Silvere Vialet-Chabrand[1,*]

Table S1. Growth traits of cucumber plants that were grown for 4 weeks after transplanting under four treatments: SN x aCO_2 (sinusoidal light + ambient CO_2), SN x eCO_2 (sinusoidal light + elevated CO_2), FL x aCO_2 (fluctuating light + ambient CO_2), and FL x eCO_2 (fluctuating light + elevated CO_2). Data represent means based on two experiments each with 3 replicate plants per experiment. *P*-value of the light pattern (L), CO_2, and their interaction (L x CO_2) are shown, with significant effects ($P < 0.1$) in bold. Different letters indicate significant differences between the treatments.

Growth traits		Treatment				*P*-value		
		SN x aCO_2	SN x eCO_2	FL x aCO_2	FL x eCO_2	L	CO_2	Lx CO_2
Leaves	Fresh weight	66.5 **ab**	65.2 **a**	61.4 **a**	74.4 **b**	0.489	0.089	**0.052**
	Dry weight	10.1	12.3	7.1	10.1	**0.008**	**0.008**	0.541
	Dry weight %	15.2	19.0	11.6	13.5	**0.017**	**0.068**	0.451
Stem	Stem diameter	13.4	13.4	12.2	13.1	0.106	0.265	0.243
	Fresh weight	39.8	41.3	38.8	45.7	0.554	0.182	0.348
	Dry weight	2.5	2.6	2.3	2.8	0.942	0.112	0.362
	Dry weight %	6.2	6.3	5.9	6.0	**0.004**	**0.062**	0.541
Petiole	Fresh weight	21.9	22.3	20.8	28.3	0.446	0.244	0.283
	Dry weight	1.4	1.3	1.0	1.5	0.452	0.175	0.112
	Dry weight %	6.2	5.9	4.8	5.2	**0.025**	0.907	0.343

Table S2. Dynamic photosynthesis parameters: steady-state A at 120 µmol m^{-2} s^{-1} light during induction (A_{120}), steady-state A at 1200 µmol m^{-2} s^{-1} light during induction (A_{1200}), induction state after 60 s (IS_{60}), time to reach 50% of IS (T_{50}), time to reach 90% of IS (T_{90}), g_s at 120 µmol m^{-2} s^{-1} light (g_{s120}), g_s at 1200 µmol m^{-2} s^{-1} light (g_{s1200}), time to reach 63% of g_s during step increase from 120 to 1200 µmol m^{-2} s^{-1} ($\tau_{induction}$), time to reach 63% of g_s during step decrease from 1200 to 120 µmol m^{-2} s^{-1} ($\tau_{relaxation}$), shape constant during step increase ($\lambda_{induction}$), shape constant during step decrease ($\lambda_{relaxation}$). Cucumber plants were grown for 4 weeks after transplanting under four treatments: SN x aCO_2 (sinusoidal light + ambient CO_2), SN x eCO_2 (sinusoidal light + elevated CO_2), FL x aCO_2 (fluctuating light + ambient CO_2), and FL x eCO_2 (fluctuating light + elevated CO_2). Data represent means of 3-5 replicate plants. ANOVA with unbalanced design was performed. *P*-values of light pattern (L), CO_2, and their interaction (L x CO_2) are shown, with significant effects ($P < 0.1$) in bold.

Parameters	Treatment				*P*-values		
	SN x aCO_2	SN x eCO_2	FL x aCO_2	FL x eCO_2	L	CO_2	L x CO_2
A_{120} (µmol m^{-2} s^{-1})	4.8	4.9	5.3	5.2	0.133	0.887	0.677
A_{1200} (µmol m^{-2} s^{-1})	20.2 b	18.1 ab	17.0 a	19.7 ab	0.267	0.781	**0.076**
IS_{60} (%)	54.5	51.9	51.6	52.2	0.675	0.821	0.708
T_{50} (s)	53.0	54.2	85.3	53.8	0.295	0.439	0.406
T_{90} (s)	439.8	343.7	533.6	331.6	0.641	0.224	0.657
g_{s120} (mol m^{-2} s^{-1})	0.25 b	0.26 b	0.14 a	0.23 b	**<0.001**	**0.011**	**0.019**
g_{s1200} (mol m^{-2} s^{-1})	0.36 b	0.34 ab	0.30 a	0.41 c	0.492	**0.026**	**0.005**
$\tau_{induction}$ (s)	730.1	780.3	627.2	626.3	**0.032**	0.643	0.632
$\tau_{relaxation}$ (s)	412.6	424.1	330.1	270.5	**0.022**	0.585	0.423
$\lambda_{induction}$	1.6 a	2.3 b	1.8 ab	1.4 a	0.318	0.588	**0.048**
$\lambda_{relaxation}$	1.3	1.3	1.1	1.0	0.188	0.915	0.885

Table S3. Key characteristics of sinusoidal (SN) and fluctuating (FL) light patterns. Data were first aggregated per day of treatment, then averaged per round of treatment; finally, averages of the four treatment rounds per light treatment were calculated.

Light pattern characteristics	SN	FL
No. of light intensity shifts per photoperiod	63.0	289.2
Maximum amplitude of greatest PPFD change (μmol m^{-2} s^{-1})	120.0	1074.0
Average amplitude (μmol m^{-2} s^{-1})	8.3	292.9
Duration at 120 μmol m^{-2} s^{-1} (h)	0.3	9.7
Duration at 121 - 400 μmol m^{-2} s^{-1} (h)	15.8	3.6
Duration at >400 μmol m^{-2} s^{-1} (h)	0.0	2.7

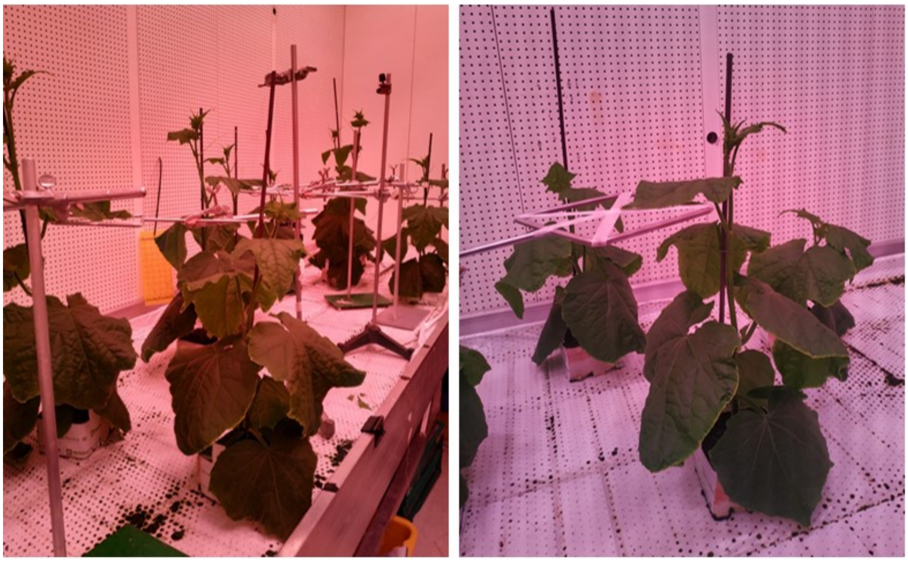

Figure S1. Leaf support structure used to make leaf#6 horizontal. While the light spectrum was identical throughout the experiment, it appears different between these two images due to automatic changes in camera settings.

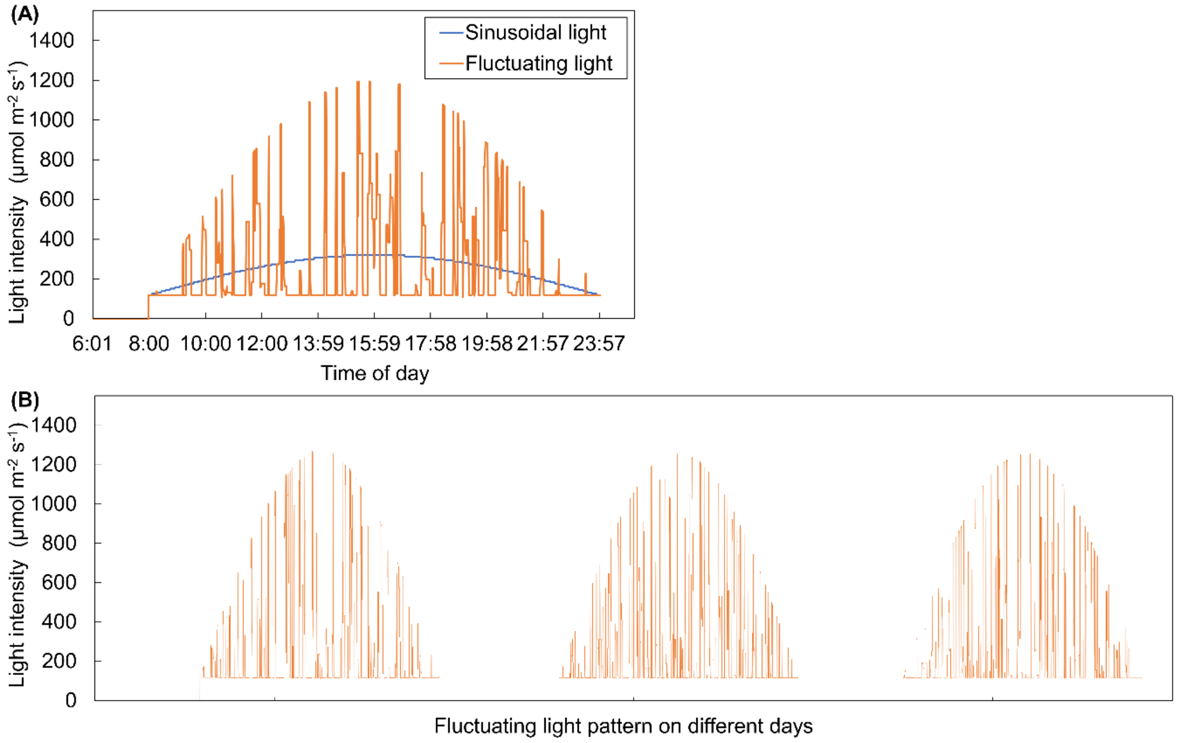

Figure S2. Light treatments used for plant growth: A) light patterns – fluctuating and sinusoidal light, B) examples of fluctuating light patterns on different days.

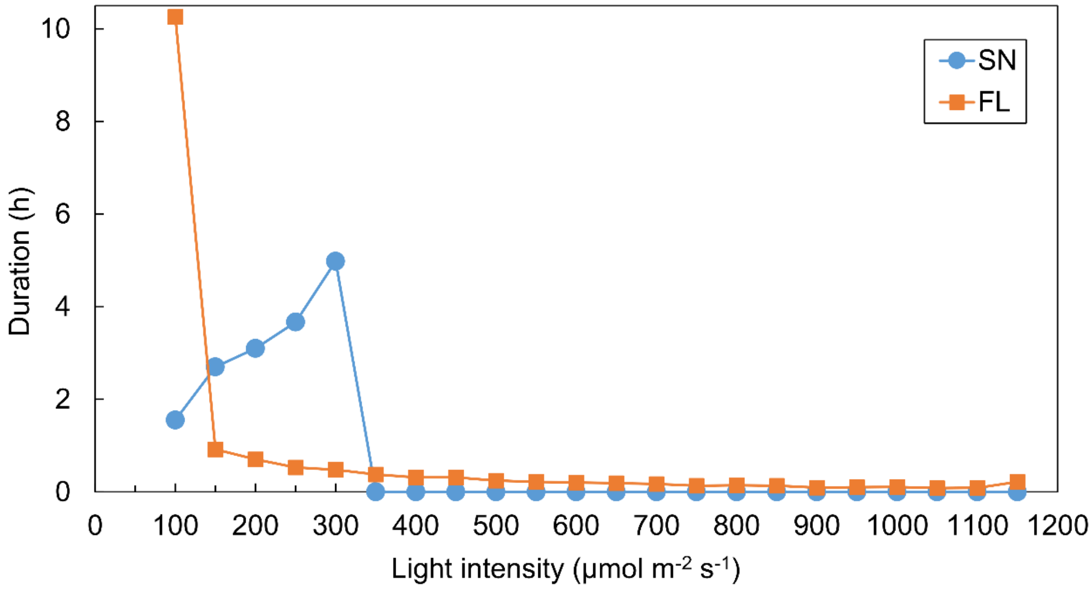

Figure S3. Duration (hours) per category of light intensity during sinusoidal (SN) and fluctuating (FL) light patterns. The light intensity categories were binned per 50 µmol m^{-2} s^{-1}, e.g. 100-150 µmol m^{-2} s^{-1}, 150-200 µmol m^{-2} s^{-1}, etc. Data were first aggregated per day of treatment, then averaged per round of treatment; finally, averages of four treatment rounds per light treatment were calculated and displayed.

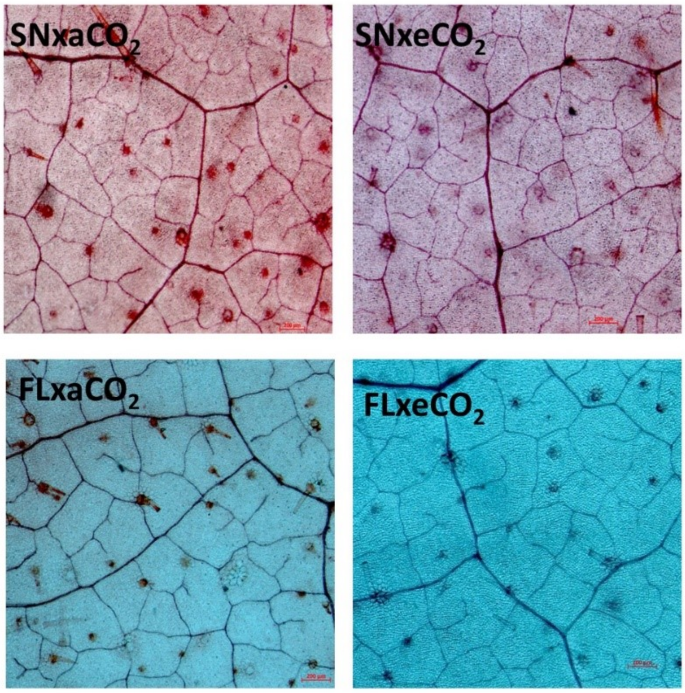

Figure S4. Images of leaf veins (excluding primary and secondary veins) of cucumber plants grown for 4 weeks after transplanting under four treatments: SN x aCO_2 (sinusoidal light + ambient CO_2), SN x eCO_2 (sinusoidal light + elevated CO_2), FL x aCO_2 (fluctuating light + ambient CO_2), and FL x eCO_2 (fluctuating light + elevated CO_2).

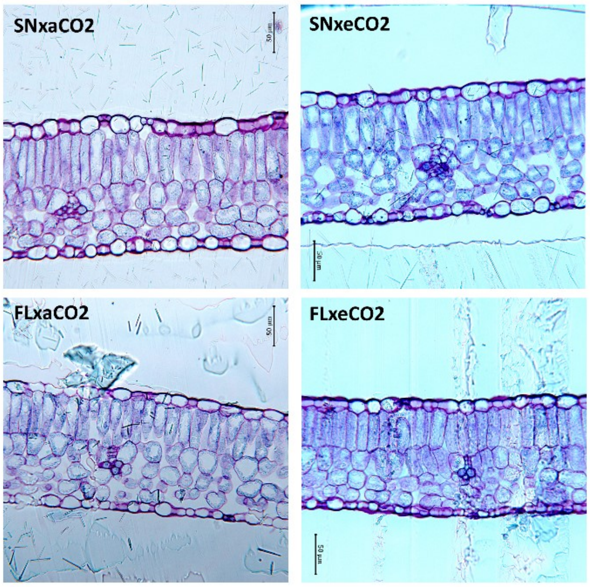

Figure S5. Leaf cross section images of cucumber plants grown for 4 weeks after transplanting under four treatments: SN x aCO_2 (sinusoidal light + ambient CO_2), SN x eCO_2 (sinusoidal light + elevated CO_2), FL x aCO_2 (fluctuating light + ambient CO_2), and FL x eCO_2 (fluctuating light + elevated CO_2).

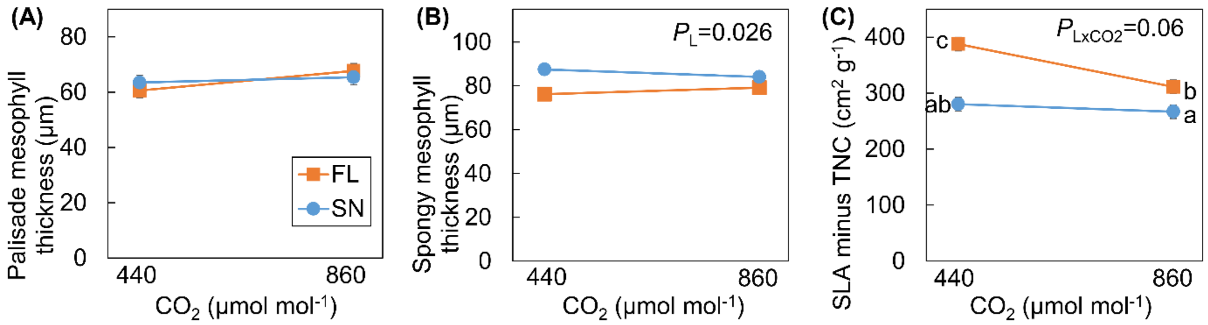

Figure S6. Leaf anatomical properties: A) palisade mesophyll length, B) spongy mesophyll length. C) specific leaf area (SLA), calculated by subtracting the total non-structural carbohydrates (TNCs; sum of glucose, fructose, sucrose, stachyose, and starch). Cucumber plants were grown under four treatments: SN x aCO_2 (sinusoidal light + ambient CO_2), SN x eCO_2 (sinusoidal light + elevated CO_2), FL x aCO_2 (fluctuating light + ambient CO_2), and FL x eCO_2 (fluctuating light + elevated CO_2). Data represent mean ± SEM based on two experiments each with 2-3 replicate plants per experiment. P-value of the main effect of light pattern (P_L) and interaction effect of light pattern and CO_2 (P_{LxCO2}) is shown, when $P < 0.1$. Different letters indicate significant differences between the treatments.

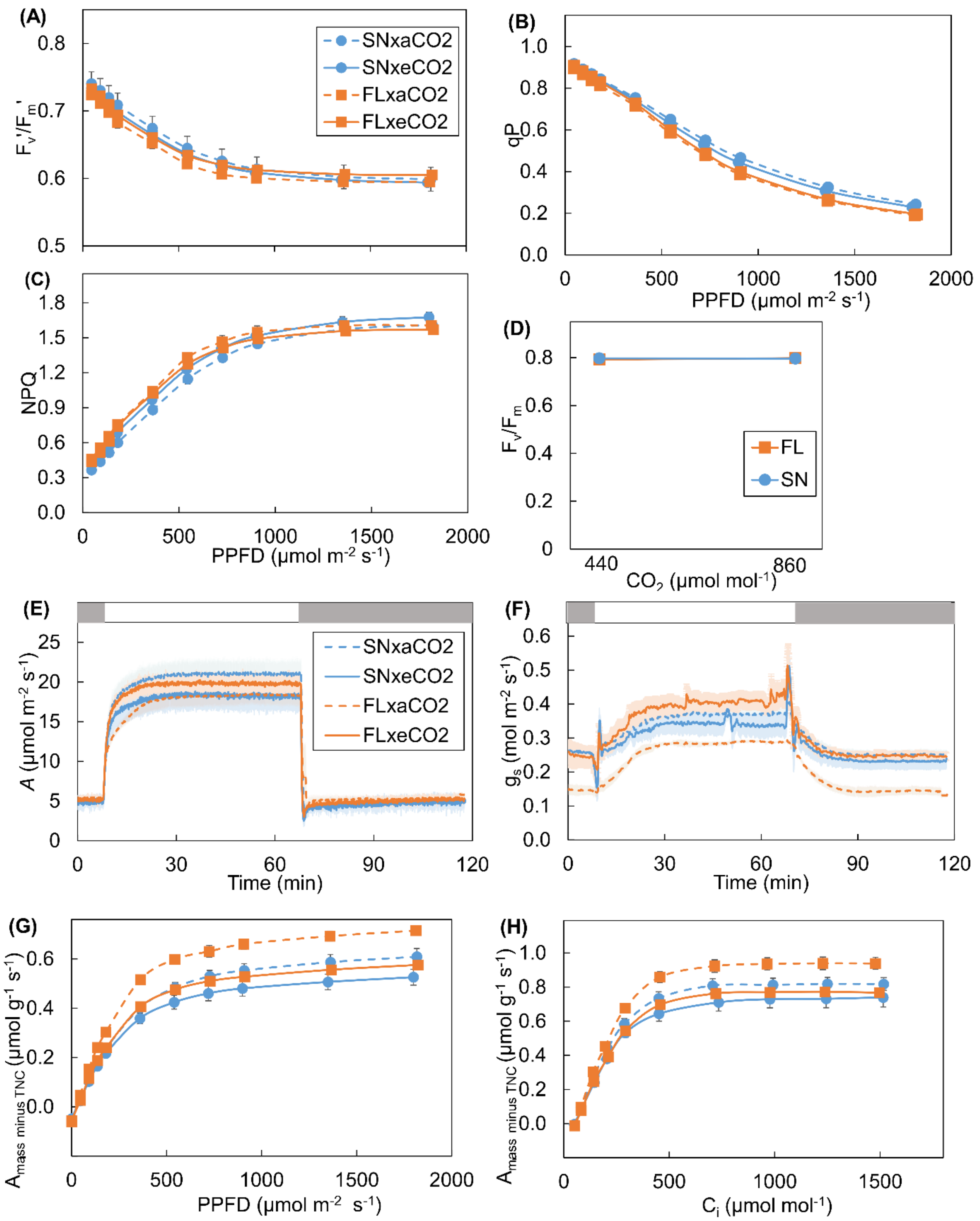

Figure S7. Steady-state and dynamic photosynthesis. Steady-state photosynthesis as a function of light absorbed by the leaf (PPFD): A) PSII maximum efficiency (F_v'/F_m'), B) PSII efficiency factor (qP), C) non-photochemical quenching (NPQ), D) maximum quantum efficiency of PSII photochemistry (F_v/F_m, measured at ca. 11.00 in the morning). Dynamic photosynthetic responses to step increase and decrease in light intensity: E) net photosynthesis rate (*A*), F) stomatal conductance (g_s); grey and white bars represent the time in low light (120 μmol m^{-2} s^{-1}) and high light (1200 μmol m^{-2} s^{-1}), respectively. Steady-state photosynthesis per unit leaf mass minus the total non-structural carbohydrates (TNCs; sum of glucose, fructose, sucrose, stachyose, and starch) as a function of: (G) PPFD, (H) intercellular CO_2

concentration (C_i). Cucumber plants were grown under four treatments: SN x aCO_2 (sinusoidal light + ambient CO_2), SN x eCO_2 (sinusoidal light + elevated CO_2), FL x aCO_2 (fluctuating light + ambient CO_2), and FL x eCO_2 (fluctuating light + elevated CO_2). Data represent means ± SEM of 5-7 plants for steady-state and 3-5 plants for dynamic photosynthesis.

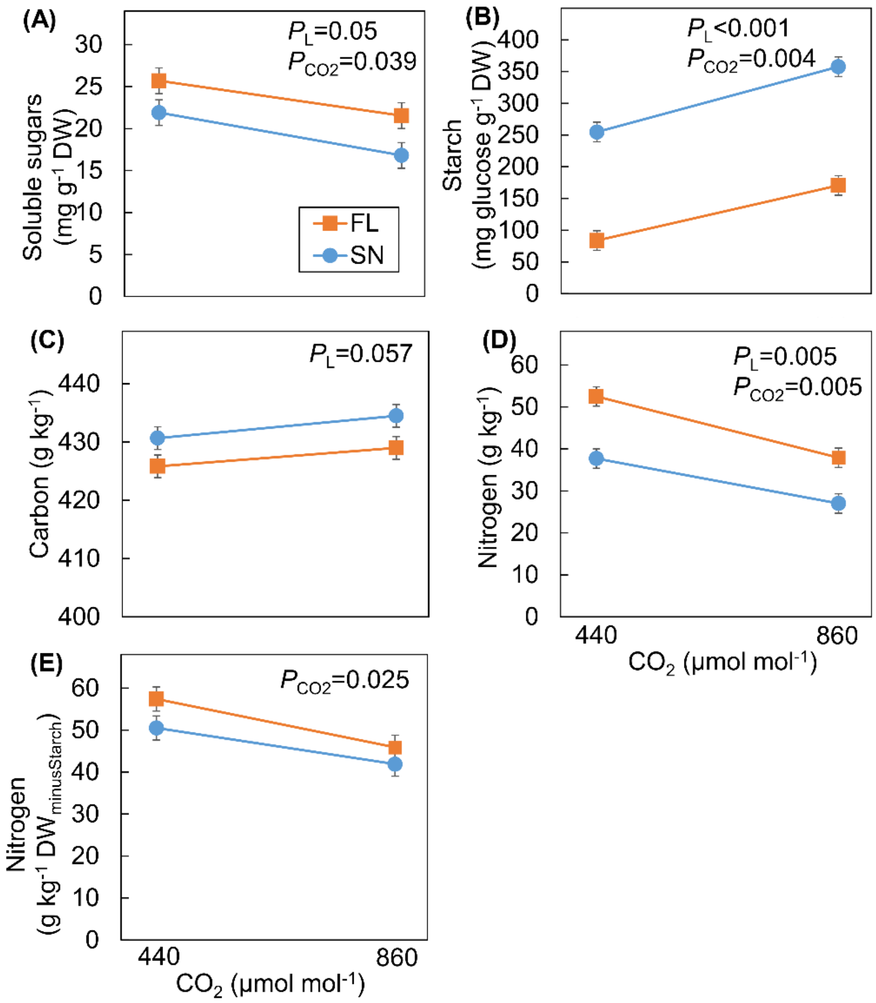

Figure S8. Leaf carbohydrate, carbon, and nitrogen content per unit leaf dry weight (DW): A) soluble sugars (sum of glucose, fructose, sucrose, and stachyose), B) starch content, C) carbon content, D) nitrogen content, (E) nitrogen content per unit DW minus starch. Cucumber plants were grown under four treatments: SN x aCO_2 (sinusoidal light + ambient CO_2), SN x eCO_2 (sinusoidal light + elevated CO_2), FL x aCO_2 (fluctuating light + ambient CO_2), and FL x eCO_2 (fluctuating light + elevated CO_2). Data represent means ± SEM based on two experiments each with 2-3 replicate plants per experiment. P-value of the main effect of light pattern (P_L) and CO_2 (P_{CO2}) is shown, when $P < 0.1$.

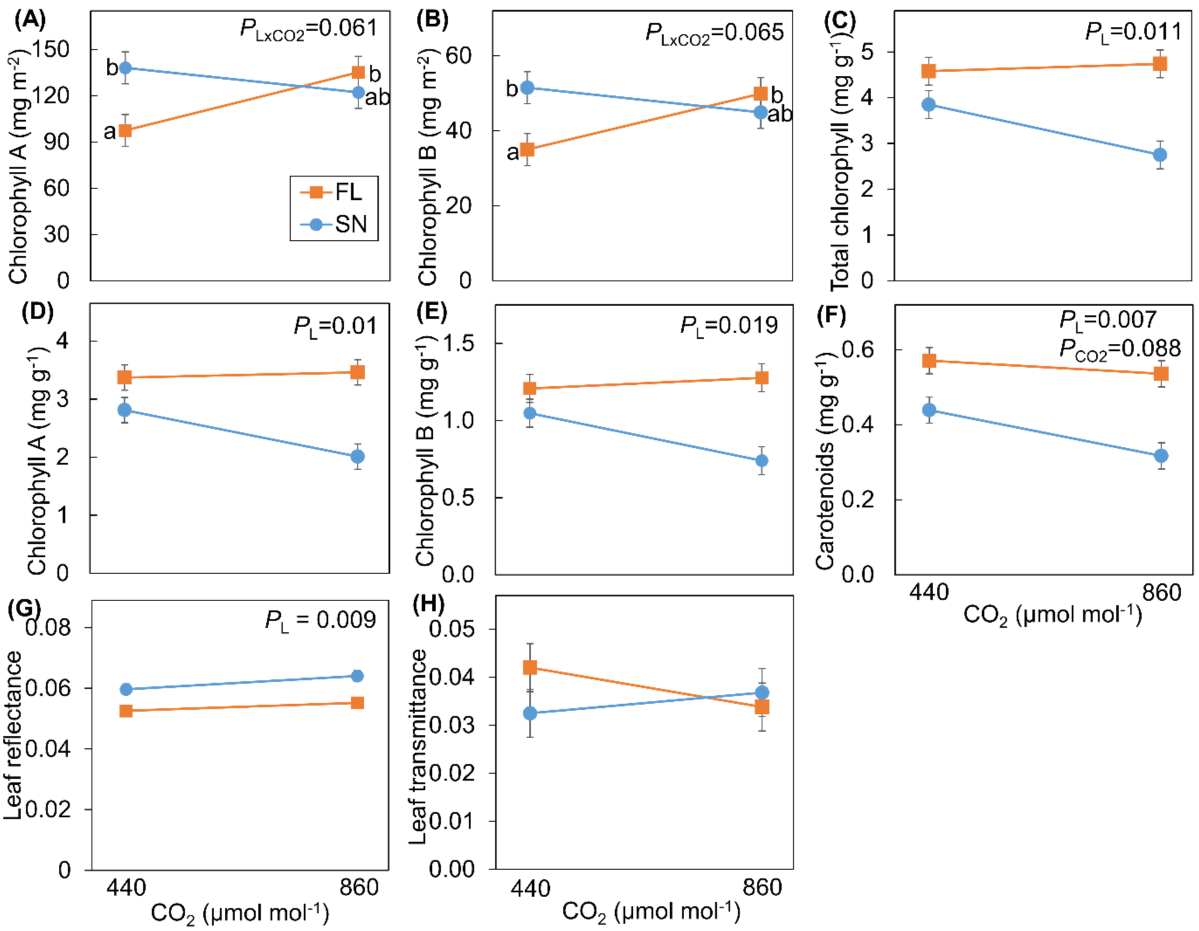

Figure S9. Leaf pigment content and optical properties: A) chlorophyll A per unit leaf area, B) chlorophyll B per unit leaf area; pigment content expressed per unit leaf dry weight: C) total chlorophyll content, D) chlorophyll A, E) chlorophyll B, F) carotenoids; G) leaf reflectance, H) leaf transmittance. Cucumber plants were grown under four treatments: SN x aCO_2 (sinusoidal light + ambient CO_2), SN x eCO_2 (sinusoidal light + elevated CO_2), FL x aCO_2 (fluctuating light + ambient CO_2), and FL x eCO_2 (fluctuating light + elevated CO_2). Data represent means ± SEM based on two experiments each with 2-3 replicate plants per experiment. *P*-value of the light pattern (P_L), CO_2 (P_{CO2}), and interaction effect of light pattern and CO_2 (P_{LxCO2}) is shown, when $P < 0.1$. Different letters indicate significant differences between the treatments.
